# Population genetic diversity of invasive *Pomacea* snails and surveillance of *Angiostrongylus cantonensis* in Shanghai, East China

**DOI:** 10.1186/s13071-025-07224-w

**Published:** 2026-03-09

**Authors:** Peter S. Andrus, Qing-chi Han, Li-min Yang, Christopher M. Wade, Zhi-qiang Qin, Kokouvi Kassegne, Xiao-nen Wu, Yun-hai Guo, Xiao-nong Zhou

**Affiliations:** 1 School of Global Health, Chinese Center for Tropical Diseases Research, Shanghai Jiao Tong University School of Medicine, Shanghai, China; 2 National Institute of Parasitic Diseases, Chinese Center for Disease Control and Prevention (Chinese Center for Tropical Diseases Research), Shanghai, China; 3 School of Life Sciences, University of Nottingham, Nottingham, UK; 4 Hainan Tropical Diseases Research Center (Hainan Sub-Center, Chinese Center for Tropical Diseases Research), Haikou, Hainan China

**Keywords:** Population genetics, Molecular xenomonitoring, *Pomacea*, *Angiostrongylus cantonensis*

## Abstract

**Background:**

Golden apple snails (Gastropoda: Ampullariidae: *Pomacea*) were introduced into China in the 1980s for aquaculture and have since become widespread agricultural pests across East Asia. In addition to their invasive impact, they are a key intermediate host of the rat lungworm *Angiostrongylus cantonensis* (Secernentea: Angiostrongylidae) in China, the causative agent of eosinophilic meningitis in humans.

**Methods:**

We conducted a malacological survey of 55 freshwater sites across Shanghai and neighboring East China provinces to assess *Pomacea* distribution, genetic diversity, and *A. cantonensis* infection status. A total of 700 *Pomacea* snails were examined for *A. cantonensis* using traditional lung microscopy and molecular xenomonitoring (PCR and LAMP). Mitochondrial *COI* barcoding was performed on 200 individuals from 20 high-density sites to assess species composition and genetic diversity.

**Results:**

*Pomacea* snails were found at 81.8% (45/55) of sites surveyed. No *A. cantonensis* infections were detected by microscopy or molecular assays. Genetic analyzes revealed three *Pomacea* species (*P. canaliculata*, *P. maculata*, and *P. occulta*) and nine distinct *COI* haplotypes. *Pomacea canaliculata* was the most common and genetically diverse species, with four unique haplotypes (H5–H8) occurring only in Shanghai, indicative of recent introductions. Overall, populations showed moderate haplotype diversity (*Hd* = 0.73) and population structure (*F*_ST_ = 0.24).

**Conclusions:**

Although no *A. cantonensis* infections were detected in the snails examined in this survey, these negative findings do not preclude the possibility of low-prevalence or newly emerging infections. The wide distribution and high genetic diversity of *Pomacea* populations across Shanghai and East China highlight that suitable hosts are already well-established, emphasizing the ongoing risk of parasite introduction and spread into currently nonendemic regions. Continued molecular surveillance, public awareness, and strengthened biosecurity measures remain essential to effectively manage invasive snail populations and mitigate future public health threats.

**Graphical abstract:**

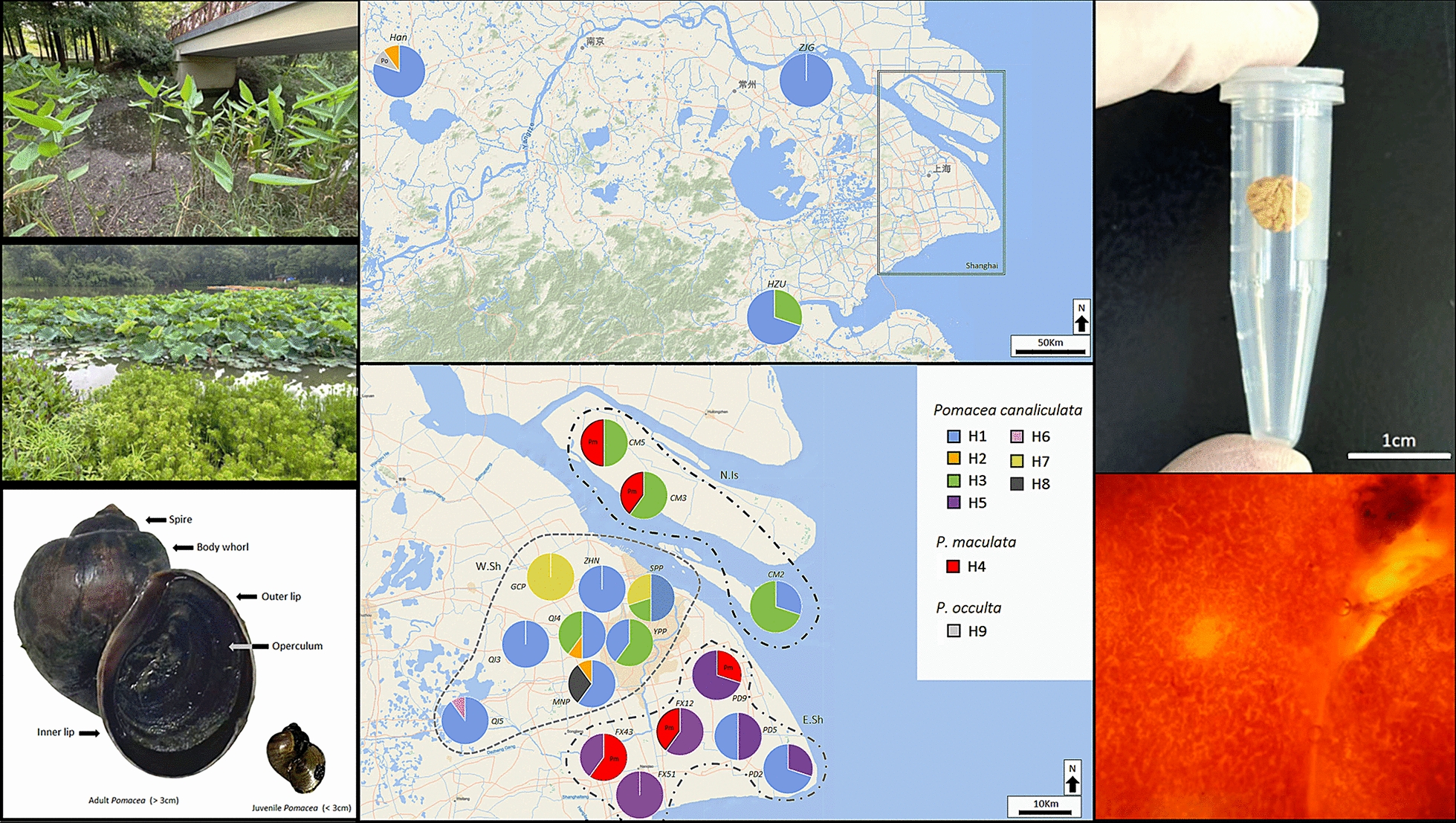

**Supplementary Information:**

The online version contains supplementary material available at 10.1186/s13071-025-07224-w.

## Background

The globalization of trade and travel has accelerated the spread of invasive species, threatening the agriculture, biodiversity, and public health of developed and developing countries [1]. Among the most problematic is the South American freshwater snail genus *Pomacea* (Perry, 1810), which has become an invasive pest in Southeast Asia, the Pacific islands, Europe, and the USA [2–4]. *Pomacea* snails were first introduced to Guangdong, China in 1981, for aquaculture, and were initially promoted as a low-cost food source [5, 6]. They rapidly spread across the southern provinces of China during the early 1980s, driven by economic interest. However, their popularity declined in the 1990s due to poor market returns and increasing reports of crop damage [7]. Intentional introductions decreased sharply, but wild populations (mainly *P. canaliculata*) had already become established due to their high fecundity, environmental resilience, and broad diet [5, 8]. Since then, *Pomacea* snails have emerged as major agricultural pests of wetland crops, particularly rice, across Southeast Asia.

Despite extensive control efforts, *Pomacea* snails continue to expand their range in China and Southeast Asia, primarily through human-mediated dispersal [7]. By 2007, *Pomacea* snails were already widespread across southern China and had reached as far north as Ningbo, Zhejiang [9]. By 2022, their distribution had expanded into northern provinces, including Shanghai, Jiangsu, Hubei, Chongqing, and Sichuan, thereby crossing the Yangtze River barrier [10]. Understanding the mechanisms behind the successful establishment of invasive *Pomacea* snails, particularly their dispersal history and population structure, is essential for designing effective control strategies. Previous genetic studies using mitochondrial and nuclear markers have documented the coexistence of multiple *Pomacea* species (*P. canaliculata*, *P. maculata*, and *P. occulta*) in China, with evidence of repeated introductions shaped by both natural dispersal and human-mediated transport [5, 6, 10, 11]. More recently, population genomic studies have identified exceptionally high genetic diversity and clear signatures of cold adaptation in *P. canaliculata* populations across Southeast Asia and China, highlighting the rapid evolutionary potential of these species [12].

In addition to their ecological impact, *Pomacea* snails are important intermediate hosts of the rat lungworm *Angiostrongylus cantonensis*, the primary cause of eosinophilic meningitis in humans [13]. Human infection occurs through the ingestion of third-stage larvae via consuming contaminated vegetables, unboiled water, or eating undercooked infected gastropods [14]. Between 1997 and 2006, approximately 75% of the 334 recorded human angiostrongyliasis cases in China were linked to consumption of *Pomacea* snails [15]. The continued spread of invasive gastropod species such as *Pomacea* has increased the risk of *A. cantonensis* transmission across China [9, 16], South Asia [2], and parts of Europe [17]. Moreover, the recent detection of *Angiostrongylus cantonensis*-infected *Pomacea canaliculata* snails in Ma’anshan City, Anhui Province [18], is a significant development, as this area lies directly adjacent to the Jiangsu Province border and the capital city of Nanjing. This finding indicates that infected intermediate hosts are now emerging in densely populated parts of eastern China, well beyond the parasite’s traditional endemic range in the south. Therefore, the effective surveillance of *A. cantonensis* in gastropod hosts is essential for preventing future outbreaks and controlling transmission. There is no definitive gold standard for diagnosing *Angiostrongylus* infection in gastropods. However, lung microscopy is widely used because larvae usually concentrate in the lung sac and can be detected by nodules [19, 20]. Similarly, molecular detection methods such as PCR and LAMP offer greater sensitivity and can detect infection in gastropods [21, 22], rats [23], and humans [24]. In this study, we examined the genetic diversity of invasive *Pomacea* populations in Shanghai and neighboring East China provinces, while also assessing the current prevalence of *A. cantonensis* infection using lung microscopy and molecular detection methods. This study aims to clarify the population structure of *Pomacea* snails in Shanghai and assess their potential role in the transmission of *A. cantonensis* in Shanghai and the East China region.

## Methods

### Sample sites, snail collection, and *Pomacea* dissection

**Fig. 1:**
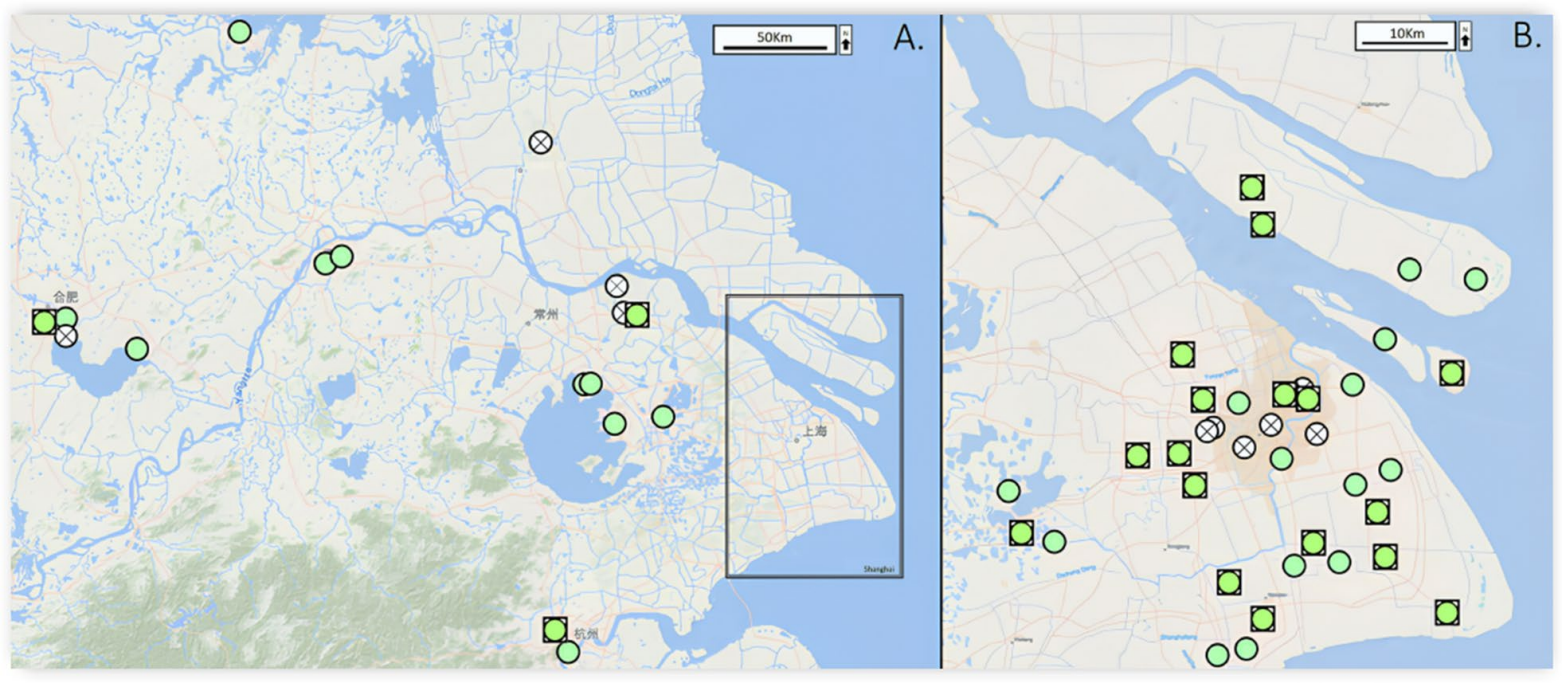
Maps of collection sites in East China (**A**) and Shanghai (**B**). White circles with a cross indicate sites where *Pomacea* snails were absent. Green circles indicate sites where *Pomacea* was present, and green squares denote the 20 high-density sites selected for genetic analysis. Maps were created using ArcGIS 10.5 (Esri, CA, USA; https://www.arcgis.com/)

All sites were surveyed once between April 2024 and November 2024, which corresponds to the main summer–autumn activity period for *Pomacea* snails in East China. In total, 55 freshwater sites across East China were checked for the presence of invasive *Pomacea* snails (Fig. 1). The study focused primarily on freshwater habitats across Shanghai (*n* = 37), with additional sampling in Anhui (*n* = 4), Jiangsu (*n* = 12), and Zhejiang (*n* = 2) to compare with and provide regional context (Supplementary Table S1). Freshwater sites were selected to represent diverse aquatic ecosystems, including popular lakes, urban parks, protected wetlands, and agricultural zones. Site selection considered the hydrological connectivity across the Yangtze River Delta, including the Huangpu River and its tributaries, as well as the Grand Canal network. Each site was surveyed for 1 h and was checked for the presence of *Pomacea* snails and their distinct, vibrant pink egg clutches (Supplementary Fig. S1A–D). When present, *Pomacea* snails were identified on the basis of standard morphological features (e.g., globose shape, deep umbilicus, and operculum), while species-level identity was later confirmed using *COI* barcoding (Supplementary Fig. S1D) [20, 25]. All adult *Pomacea* snails (> 3.5 cm shell length) observed within 1 h of searching were hand-collected for later dissection. Many *Pomacea* populations in East China are newly established and had low densities of *Pomacea* snails, making it difficult to consistently quantify snails per square meter. Instead, sites were classified as high density if more than ten adult snails were present, and as low density if only juveniles and/or egg clutches were found. All collected snails were transported to the National Institute of Parasitic Diseases, China CDC, Shanghai for subsequent dissection and DNA extraction.

After collection, all adult *Pomacea* snails were stored at 5 °C and dissected within 48 h by a trained team from the *Angiostrongylus* Department of the National Institute of Parasitic Diseases, China CDC, to assess potential *A. cantonensis* infection. Cooling at 5 °C was used solely as an immobilization step to reduce activity and handling stress. Prior to dissection, loss of responsiveness was confirmed by the absence of withdrawal reflexes following tactile stimulation of the foot. Next, snails were rinsed with tap water to remove external debris. The operculum was then detached by sliding a scalpel between the inner lip and the operculum to sever the connecting tissue. The shell was opened by cutting along the body-whorl suture from the outer lip toward the spire using dissecting scissors, after which the soft body was removed following the procedures described in Zhao et al. [20]. Euthanasia was then performed by rapid destruction of the central ganglion using a scalpel, resulting in immediate loss of neural function. Next, the hepatopancreas, hemolymph, and lung sac were examined for juvenile *A. cantonensis* nematodes. In particular, the lung sac was carefully opened and inspected under a dissection microscope for infection-related nodules using the lung microscopy method (as described in Zhao et al. [20]; Supplementary Fig. S1E). Lung microscopy is preferred for diagnosing *A. cantonensis* infection in large numbers of *Pomacea* snails due to its time efficiency and the tendency of larvae to aggregate in the pulmonary cavity (lung sac), rather than in the muscular foot or digestive tract [19, 26]. Each tissue sample was classified as either normal (smooth, translucent tissue), abnormal (irregular or discolored tissue), or nodule-present (contains small, spherical nodules ∼0.25 mm in diameter with no surrounding translucent material), based on criteria agreed upon by the dissection team. Following microscopic inspection, a circular tissue sample (7 mm diameter; 38.5 mm^2^) was excised from each lung sac using a sterile borer (Supplementary Fig. S1F). All abnormal or nodule-bearing samples were subjected to molecular detection for possible *A. cantonensis* infection. If all lung samples from a site appeared normal, a subset of 20 randomly selected samples from that site was tested instead. Lung tissue samples were preserved in 95% ethanol and stored at −20 °C for subsequent DNA extraction. Further information on *A. cantonensis* infection screening can be found in Supplementary Table S2.

### *Pomacea* lung DNA extraction

Following collection and dissection, preserved lung tissue samples were subjected to DNA extraction using a modified CTAB (Cetyl-Trimethyl-Ammonium Bromide) protocol, as described by Goodacre and Wade [27]. In brief, 20 lung tissue samples per site were individually air-dried and homogenized in 500 µL of CTAB extraction buffer (100 mM Tris–HCl, 20 mM EDTA, 1.4 M NaCl, 2% CTAB), 10 µL of Proteinase K, and 1 µL of β-mercaptoethanol. Samples were incubated at 56 °C for 2 h with intermittent vortexing until complete digestion. Next, 500 µL of chloroform-isoamyl alcohol (24:1) was added, and samples were gently inverted for 5 min and centrifuged at 13,000 rpm for 5 min. The upper aqueous layer was carefully transferred to a new Eppendorf tube, and the chloroform extraction was repeated once to improve purity. The DNA was precipitated by adding 1 mL of cold 95% ethanol and 4 µL of 3 M sodium acetate, followed by centrifugation at 13,000 rpm for 10 min. The supernatant was discarded, and the resulting DNA pellet was air-dried and resuspended in 200 µL of TE buffer (10 mM Tris–HCl, pH 8.0). The DNA extracts were stored at −20 °C for subsequent *COI* genotyping and molecular xenomonitoring.

### PCR and LAMP infection detection

After DNA extraction, the *Pomacea* DNA samples were tested for *A. cantonensis* infection targeting the Internal Transcribed Spacer 2 (*ITS2*) using the Anc-*ITS2* primers as described by Jiang et al. [19] (Supplementary Table S3). First, 20 DNA extracts per site were selected and diluted to 50 ng/µL using ddH_2_O. All PCR reactions were performed using a 25 μL reaction volume containing 24 μL of Tiangen PCR master mix (1U TAQ, 0.2 μM primers, 200 μM dNTP, 1.5 mM MgCl_2_), and 1 µL of DNA template. To confirm PCR specificity, we tested the Anc-*ITS2* primers against other gastropod-associated nematodes (*Caenorhabditis*, Mermithidae sp., *Oscheius*, Panagrolaimidae sp., *Phasmarhabditis*, and *Rhabdias*) to ensure that they did not cross-amplify nontarget species that could exist in wild *Pomacea* snails (Supplementary Fig. S2). To confirm the absence of nonspecific amplification, each PCR run included a no-template control (water), an uninfected *Pomacea* DNA control, a positive control containing pure *A. cantonensis* DNA, and an infected *Pomacea* DNA control. The PCR cycling conditions used were an initial denaturation at 94 °C for 5 min, followed by 35 cycles of denaturation at 94 °C for 30 s, annealing at 50 °C for 30 secs, and extension at 72 °C for 60 s, with a final extension at 72 °C for 10 min. PCR products were run on a 1.2% agarose gel stained with GelStain Red (1000X) and visualized under UV light. Nonspecific faint bands were occasionally observed at the initial annealing temperature (50 °C). To eliminate nonspecific products, the PCR was repeated and the annealing temperature was increased to 55 °C (Supplementary Fig. S3).

Following the PCR-based infection detection, if a site had an Anc-*ITS2* PCR positive result, then all samples would be tested using an *A. cantonensis* specific LAMP assay, using newly designed Anc-*COI* primers. The Anc-*COI* LAMP primers were designed using the mitogenomic sequences of several *A. cantonensis* mitogenomes (GQ398121, KT947978, MK570632, OR177659, and PP748571) retrieved from the NCBI GenBank database. Primer specificity was validated in silico against mitochondrial genomes of closely related Metastrongyloidea species, including *A. malaysiensis* (NC_030332), *A. costaricensis* (NC_013067), and *Necator americanus* (NC_003416), confirming no significant matches with nontarget species. LAMP primer design was carried out using the NEB LAMP Primer Design Tool v1.4.2 (https://lamp.neb.com/#/) to generate a complete set of four primers (F3, B3, FIP and BIP; Supplementary Fig. S4; Supplementary Table S3). All primers were synthesized by Sangon Biotechnology Co., Ltd. (Shanghai, China). The Anc-*COI* LAMP reaction was conducted in a total volume of 25 µL using New England Biolabs Bst 2.0 (M0537L). The reaction mixture contained 12.5 µL of buffer mix (0.8 mM MgSO_4_, 1.4 mM dNTPs, and 0.8 M betaine), 1 µL of Primer Master Mix (FIP 1.6 µM, BIP 1.6 µM, F3 0.2 µM, B3 0.2 µM and LB 0.4 µM), 1 µL of Bst 2.0 DNA polymerase, 2 µL of DNA template, and 8.5 µL of nuclease-free water. All LAMP reactions were incubated at 65 °C for 60 min in a LA-500 Loopamp real-time turbidimeter (Eiken Chemical Co., Ltd., Tochigi, Japan), followed by an inactivation step at 80 °C for 5 min. LAMP amplification was monitored using real-time turbidity detection. Each run included full positive and negative controls to verify assay performance, and contamination was minimized through strict separation of pre- and postamplification areas, the use of filter tips, and dedicated clean workstations. Samples were considered *A. cantonensis*–positive when turbidity exceeded 0.4, which represented the minimum consistent signal produced by our calibrated positive controls.

### *Pomacea COI* genotyping and population genetics analysis

Genomic DNA samples from 20 sites across East China were selected to measure the genetic diversity and population structure of *Pomacea* snails. Selected sites had a minimum of ten adult snails (> 3.5 cm) present and had a minimum distance of 5 km between them (Fig. 1). From each of the 20 sites, 10 random *Pomacea* DNA extracts were chosen for population genetic analysis. Population genetic analysis was performed using Cytochrome Oxidase subunit 1 (*COI*) genotyping using the universal invertebrate *COI* primers designed by Folmer et al. [28] (Supplementary Table S3). All PCR reactions were performed using a 25 μL reaction volume containing 24 μL of Tiangen PCR master mix (1U TAQ, 0.2 μM primers, 200 μM dNTP, 1.5 mM MgCl_2_) and 1 µL of DNA template. The PCR cycling conditions used for the *COI* primer sets were an initial denaturation at 96 °C for 1 min, followed by 34 cycles of 94 °C for 1 min, 50 °C for 30 s, 72 °C for 45 s and a final extension at 72 °C for 10 min. PCR products were run on a 2% agarose gel stained with GelStain Red (1000X) and visualized under UV light. The PCR products were purified and sequenced using Beijing QingKe Biotechnology Company Ltd. (Shanghai, China).

The *COI* gene was selected for analysis owing to its faster evolutionary rate, higher nucleotide diversity, and greater phylogenetic resolution compared to other genetic markers. These properties make it particularly effective for tracing the origin, migration, and dispersal patterns of geographically close populations [5, 10]. To better assess the genetic structure, the Shanghai municipality was divided into three geographic sub-populations based on major natural boundaries, which included: West Shanghai (W. Sh), comprising sites located west of the Huangpu River; East Shanghai (E. Sh), comprising sites located east of the Huangpu River; and the Northern Islands (N. Is), which included Chongming Island and Hengsha Island. This sub-regional grouping was applied to all subsequent genetic diversity and population structure analyzes to account for potential spatial barriers to gene flow and isolate patterns of localized haplotype distribution. All of the *Pomacea COI* sequences were aligned using the Multiple Sequence Comparison by Log-Expectation (MUSCLE) algorithm in BioEdit v5.0.9 [29], with any misalignments manually corrected. To determine *Pomacea* species, a phylogenetic tree was constructed using the Maximum Likelihood method in MEGA v11 [30]. The tree used a General Time Reversible model incorporating gamma correction (GTR + Γ), and a bootstrap analysis was undertaken using 1000 replicates. To measure genetic distance, pairwise distances of the *COI* haplotypes were calculated with MEGA 11 using the Maximum Composite Likelihood method. To assess the intraspecific genealogical relationships of the *COI* haplotypes, a Median-Joining (MJ) network [31] was created using the software NETWORK v5 (Fluxus Technology Ltd. www.Fluxus-engineering.com) Genetic diversity indices including the number of haplotypes (h), segregating sites (S), haplotype diversity (Hd), mean pairwise differences (K), and nucleotide diversity (π) were calculated using DnaSP v6.12 [32]. The number of defined haplotypes was confirmed by checking the nucleotide base quality scores for each of the segregating sites using FinchTV v1.4 [33]. Population structure among populations was assessed using Wright’s fixation index (F_ST_) in DNASP6 [34]. Genetic differentiation between populations was evaluated by calculating pairwise fixation indices (F_ST_), interpreted according to Wright’s criteria: low (< 0.05), moderate (0.05–0.15), high (0.15–0.25), and very high (> 0.25). Gene flow was expressed as the number of migrants per generation (Nm). Further information on the *COI* haplotypes sequenced can be found in Supplementary Table S4.

### GenBank accessions

GenBank accession numbers for the *Pomacea COI* sequences used in the phylogenetic and network analyzes can be found in Supplementary Table S5. The *COI* haplotype sequences generated in this study are available in GenBank accession numbers PV793382–PV793390.

## Results

### *Pomacea *abundance and *A. cantonensis* infection prevalence

In total, 55 East China sites were surveyed with *Pomacea* snails being present at 45 (81.8%; Fig. 1). Of those 45 sites, 20 sites had a high density of *Pomacea* snails (44.4%), while the remaining sites had a low density of *Pomacea* snails (< 10 adult snails and/or consisting mostly of egg clutches). When partitioned by province, Shanghai had the highest number of sites with 37, followed by Jiangsu with 12, Anhui with 4, and Zhejiang with 2 (Supplementary Table S1). A total of 700 *Pomacea* snails were collected from 20 sites across East China, with an average of 35 (± 25.5) snails collected per site.

When dissected, no nematodes were recovered from the hepatopancreas, hemolymph, lung sac, or surrounding tissues of any snail. In addition, no visible signs of *A. cantonensis* infection were observed in the interior of the lung sac in any of the 700 snails examined (Supplementary Table S2). However, 10 individuals from the Fengxian, Minhang, and Pudong New Area districts exhibited abnormal lung structures (Supplementary Table S2), indicating the possibility of failed or prepatent infection. To investigate this, the DNA extracts from 348 individuals were screened for infection using *A. cantonensis*-specific *ITS2* PCR primers. Of the 348 samples tested, five were PCR positive (GCP: 2/18 and MHP: 3/20; Supplementary Fig. S3A/B). However, all five positive samples had band sizes that were smaller than the expected 700 bp diagnostic band produced by the *A. cantonensis* positive controls. When the PCR was repeated, and the annealing temperature was increased from 50 °C to 55 °C, only the positive controls yielded a diagnostic band, while all field samples were negative (Supplementary Fig. S3A/B). To confirm the presence or absence of *A. cantonensis* DNA in the five suspected samples, a LAMP assay targeting the mitochondrial *COI* gene (Anc-*COI*) was performed on all samples from the Gucun park and Minhang park sites. When tested, no amplification was observed in any of the Gucun (0/18) or Minhang (0/20) samples. The only positive result was the *A. cantonensis* positive control, which produced a clear turbidity signal (maximum turbidity = 0.49; Supplementary Fig. S3C). These results indicate that the initial low-temperature PCR bands were likely false positives due to nonspecific amplification. Therefore, no *A. cantonensis* infections were confirmed in any of the collected *Pomacea* snails, based on both lung microscopy (0/700) and molecular detection methods (0/348).

### Genetic diversity of *Pomacea* populations

**Fig. 2:**
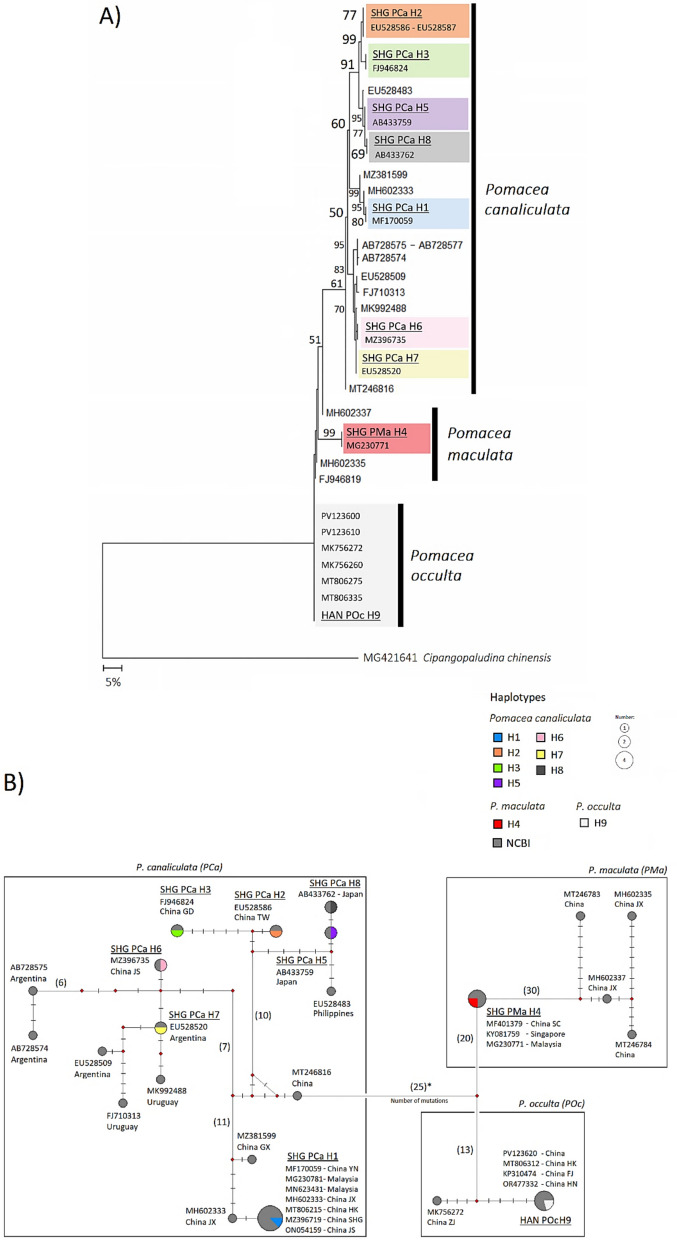
Phylogenetic and haplotype network of *Pomacea* snails from East China based on the mitochondrial *COI* gene fragment (564 bp). **A** Maximum likelihood tree (GTR + Γ model), rooted on *Cipangopaludina chinensis*. The colors represent distinct *COI* haplotypes; haplotypes from this study are bold and underlined. Numbers on branches indicate bootstrap support (%) values based on 1000 replicates (values < 50% are not shown). Scale bar = 5% nucleotide divergence. **B** Median-Joining haplotype network (NETWORK v5). Circles represent haplotypes, with size proportional to the number of individuals. Diamonds represent inferred intermediate haplotypes. Hatch marks show nucleotide substitutions between haplotypes (values > 5 are shown numerically). NCBI reference sequences are shown in dark grey with collection locations. Detailed information for all *COI* sequences used in this study is provided in Supplementary Table S5. China provinces and islands codes: FJ: Fujian; GD: Guangdong; GX: Guangxi; HAN: Hefei (Anhui); HK: Hong Kong; HN: Hainan; JS: Jiangsu; JX: Jiangxi; SC: Sichuan; SHG: Shanghai; TW: Taiwan; YN: Yunnan; ZJ: Zhejiang

Of the 700 *Pomacea* snails collected from East China, a subset of 200 individuals was selected to assess the genetic diversity of Shanghai and the surrounding East China sites. Three species were identified among them: *P. canaliculata* (*n* = 177), *P. maculata* (*n* = 22), and *P. occulta* (*n* = 1; Fig. 2A). Nine mitochondrial *COI* haplotypes (H1–H9) were detected, with *P. canaliculata* having seven (H1–H3, H5–H8), while *P. maculata* (H4) and *P. occulta* (H9) each had a single haplotype. A median-joining haplotype network revealed that the seven *P. canaliculata* haplotypes formed three clusters (H1; H2–H3, H5, H8; and H6–H7), as they were all separated by substantial nucleotide substitutions (Fig. 2B). When comparing our haplotypes with the NCBI reference sequences, we found that haplotype H1 has been widely recorded across Malaysia as well as West, South, and East China, indicating broad geographic dispersal in Southeast Asia [4, 35–38] (Fig. 2B). Likewise, the haplotypes H2–H3, H5, and H8 have been previously reported in South China, the Philippines, and Japan, suggesting regional dispersal within East and Southeast Asia [4, 39]. In contrast, haplotypes H6 and H7 were genetically similar to *P. canaliculata* sequences from Argentina and Uruguay (Fig. 2B), which represent the species’ native range [4, 40–42]. This suggests that some *P. canaliculata* populations in East China may originate from direct introductions derived from South American source populations. The single *P. maculata* haplotype (H4) grouped with sequences reported from West China, Malaysia, and Singapore, consistent with prior records [43–45]. The single *P. occulta* haplotype (H9) matched with sequences from South and East China, consistent with its known regional occurrence [11, 35, 46].

When comparing pairwise distances using the Maximum Composite Likelihood method, we found an overall genetic divergence range of 0.1–8.5% (0.001–0.085) across all *Pomacea* haplotypes (Table 1). Among the three species, the *P. canaliculata* haplotypes differed from *P. occulta* by 7.4–7.7% (0.074–0.077), and from *P. maculata* by 8.1–8.5% (0.081–0.085). The genetic distance between *P. maculata* and *P. occulta* was 4.1% (0.041). When focusing on only the *P. canaliculata* haplotypes, all seven haplotypes exhibited a divergence range of 0.1–3.6% (0.001–0.036). Haplotype H1 showed the highest divergence from both haplotypes H3 and H8 (3.60% and 3.58%, respectively), and the lowest difference from haplotypes H6 and H7 (2.82%; Table 1).

**Table 1:** Pairwise genetic distances between the *Pomacea* mitochondrial *COI* haplotypes (623 bp) using the Maximum Composite Likelihood method. Values represent the proportion of nucleotide differences between haplotypes

	H1 (Pca)	H2 (Pca)	H3 (Pca)	H4 (Pma)	H5 (Pca)	H6 (Pca)	H7 (Pca)	H8 (Pca)	H9 (Poc)
H1 (Pca)	–	0.032	0.036	0.081	0.035	0.028	0.028	0.036	0.075
H2 (Pca)	0.032	–	0.009	0.081	0.009	0.028	0.028	0.010	0.074
H3 (Pca)	0.036	0.009	–	0.081	0.013	0.033	0.033	0.015	0.077
H4 (Pma)	0.081	0.081	0.081	–	0.085	0.080	0.080	0.083	0.041
H5 (Pca)	0.035	0.009	0.013	0.085	–	0.032	0.032	0.001	0.076
H6 (Pca)	0.028	0.028	0.033	0.080	0.032	–	0.002	0.033	0.076
H7 (Pca)	0.028	0.028	0.033	0.080	0.032	0.002	–	0.033	0.077
H8 (Pca)	0.036	0.010	0.015	0.083	0.001	0.033	0.033	–	0.077
H9 (Poc)	0.075	0.074	0.077	0.041	0.076	0.076	0.077	0.077	–

Pca = *P. canaliculata*, Pma = *P. maculata*, and Poc = *P. occulta*

**Fig. 3:**
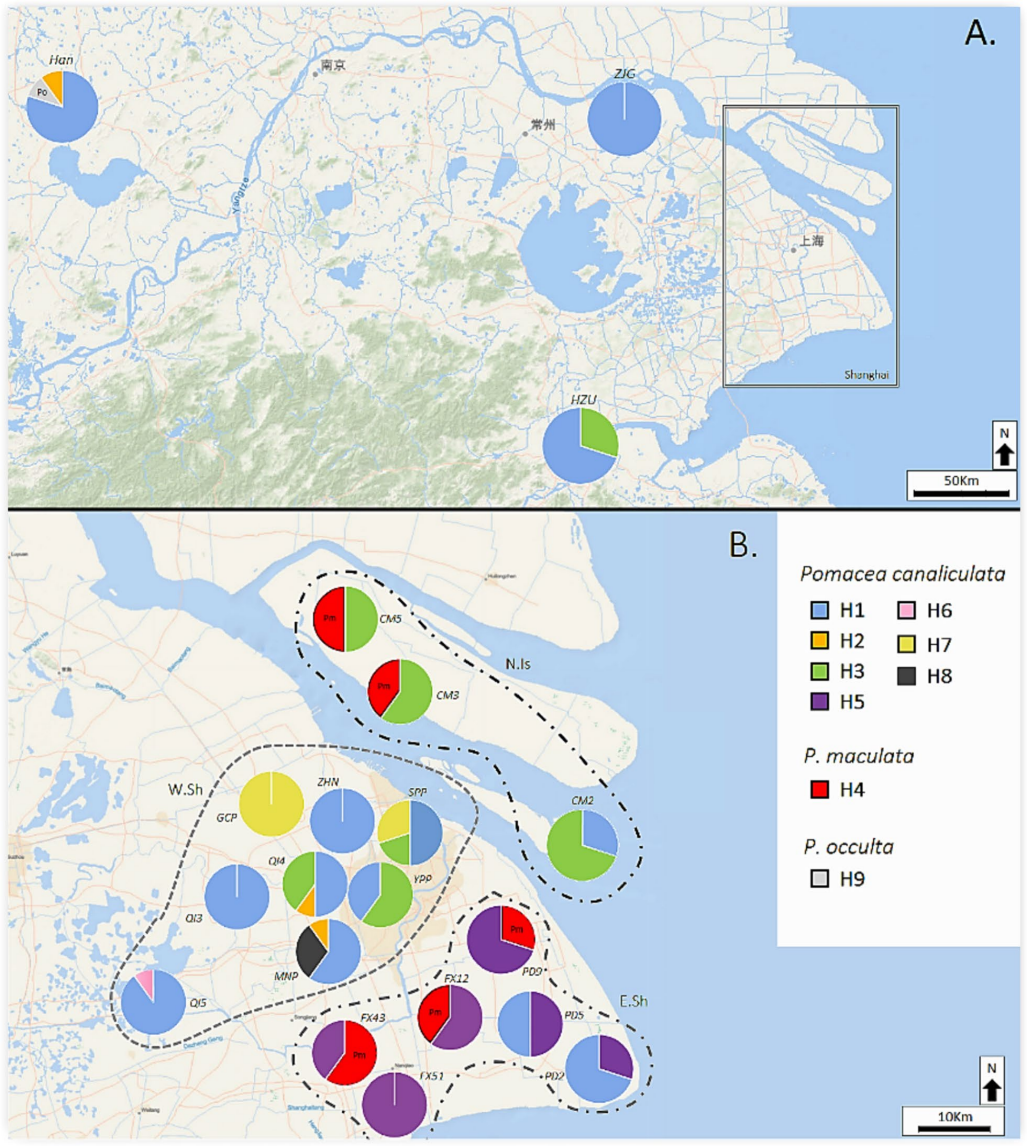
Geographic distribution of mitochondrial *COI* haplotypes (623 bp) found in *Pomacea* snails collected across East China (**A**), and detailed distribution within Shanghai (**B**). Each site (*n* = 10) represents the relative frequency of each haplotype. Shanghai sub-populations are as follows, West Shanghai (W. Sh), East Shanghai, (E. Sh) and Northern Islands (N. Islands)

Among the 20 surveyed sites, *P. canaliculata* was the most widespread species, occurring at all locations (Fig. 3). *Pomacea maculata* (H4) was restricted to East Shanghai and Northern Island populations (5 out of 20 sites), while *P. occulta* (H9) was found only at a single site in Hefei, Anhui (HAN). Overall, haplotype H1 was the most dominant, representing 44.5% of all samples (89/200). It was found in Anhui, Jiangsu, Zhejiang, and most of the Shanghai populations (10/17). Other common haplotypes showed more localized distributions, with H5 being exclusive to East Shanghai, H7 being exclusive to West Shanghai, H2 being found in both Shanghai and Anhui, and H3 being found in both Shanghai and Zhejiang. In contrast, two private haplotypes were observed only at single sites (H6 at QI5, and H8 at MNP) in Shanghai, suggesting recent mutations or localized introductions. Moreover, 5 of the 20 sites examined were made up of a singular haplotype (monomorphic), with the remaining sites having two (11/20) or three (4/20) haplotypes present. Further information on haplotype distribution at each site can be found in Supplementary Table S4.

When examining genetic diversity across all *Pomacea* species from the 20 sampled populations, overall diversity was high, with 96 segregating sites (S), a haplotype diversity (Hd) of 0.73, average pairwise nucleotide differences (K) of 25.86, nucleotide diversity (π) of 0.04, and Theta (Θ) of 0.03 (Table 2A). Haplotype diversity ranged from 0.00 (Jiangsu) to 0.77 (all Shanghai sites), while nucleotide diversity ranged from 0.00 (Jiangsu) to 0.05 (Northern Islands of Shanghai). Notably, Shanghai exhibited the highest overall genetic variation, with particularly high diversity observed in the Eastern (*Hd* = 0.58, *K* = 28.11) and Northern Island (*Hd* = 0.56, *K* = 29.79) sub-populations. Neutrality tests showed no significant departures across East China populations (Table 2A). This suggests that selection or demographic shifts, if present, are likely localized rather than widespread. However, Fu’s Fs values were elevated in several populations, especially in Shanghai (53.52), Eastern Shanghai (47.50), and the Northern Islands (33.70), consistent with potential population expansion or selection. Fu and Li’s D and F tests had significantly positive values (*P* < 0.02) in Zhejiang, Shanghai, and all three Shanghai sub-populations, indicating an excess of intermediate-frequency alleles, possibly due to balancing selection or underlying population structure (Table 2A). Tajima’s D was significantly positive in Shanghai overall (*P* < 0.01) and in the Eastern sub-population (*P* < 0.05), indicating non-neutral evolutionary patterns. In contrast, the Anhui population exhibited significantly negative values for Fu’s Fs (*P* < 0.02), Fu and Li’s D and F (*P* < 0.01), reflecting an excess of low-frequency variants indicative of recent population expansion or a founder effect.

**Table 2:** Summary of genetic diversity statistics of the East China populations by province (*n*), including all *Pomacea* species (**A**) and only *Pomacea canaliculata* (**B**) using the mitochondrial *COI* gene fragment (623 bp)

(A) All	*H*	*S*	*Hd*	*K*	*π*	Fu’s F	Fu and Li’s		Tajima’s D	Θ
							D	F		
Anhui (10)	3	68	0.378	15.98	0.026	10.69	−1.76	−1.99	−1.74*	0.039
Jiangsu (10)	1	0	0	0	0	–	–	–	–	–
Zhejiang (10)	2	30	0.466	14.0	0.022	13.61	1.59**	1.78**	1.54	0.017
Shanghai (170)	8	85	0.766	27.27	0.044	53.52	2.43**	2.83**	2.28*	0.025
W. Sh (80)	6	49	0.581	15.33	0.025	25.85	1.92**	2.15**	1.59	0.016
E. Sh (60)	3	74	0.582	28.11	0.045	47.50	2.14**	2.69**	2.41*	0.026
N. Is (30)	3	74	0.559	29.79	0.048	33.70	1.88**	2.31**	2.09*	0.031
Total (200)	9	96	0.731	25.86	0.042	51.91	0.558	1.178	1.470	0.028

(B) P. Ca	*H*	*S*	*Hd*	*K*	*π*	Fu’s F	Fu and Li’s		Tajima’s D	Θ
							D	F		
Anhui (9)	2	27	0.222	6.00	0.001	8.01	−2.25**	−2.45**	−1.97***	0.016
Jiangsu (10)	1	0	0	0	0	–	–	–	–	–
Zhejiang (10)	2	30	0.466	14.0	0.022	13.61	1.59**	1.78**	1.54	0.017
Shanghai (148)	7	49	0.712	17.93	0.029	36.59	2.03**	2.90**	2.93***	0.014
W. Sh (80)	6	49	0.581	15.33	0.025	25.85	1.92**	2.15**	1.59	0.016
E. Sh (47)	2	29	0.389	11.27	0.018	26.37	1.80**	2.39**	2.39*	0.012
N. Is (21)	2	30	0.257	7.71	0.012	14.60	1.66**	1.25	–0.29	0.013
Total (177)	7	49	0.671	17.26	0.027	38.06	2.05**	2.89**	2.85***	0.014

Shanghai sub-populations are as follows, West Shanghai (W. Sh), East Shanghai, (E. Sh), and Northern Islands (N. Is). Population-specific diversity indices include: H: number of haplotypes; S: number of segregating sites; Hd: haplotype diversity; K: mean number of pairwise nucleotide differences; π: nucleotide diversity (JC); and Ɵ (Theta): Watterson’s theta per site. Values are provided for individual provinces and sub-populations within Shanghai, with additional neutrality test statistics including Fu’s Fs, Fu and Li’s D and F, where available

Significance = *P* < 0.05*; *P* < 0.02**; *P* < 0.01***

When considering *P. canaliculata* alone, genetic diversity remained moderate-to-high, with 49 segregating sites (S), Hd of 0.67, K of 17.26, π of 0.027, and Θ of 0.014 (Table 2B). Shanghai still displayed the highest genetic variation, with the Western sub-population (*Hd* = 0.58, *K* = 15.33) being the most diverse, while Eastern Shanghai (*Hd* = 0.39, *K* = 11.27), Northern Islands (*Hd* = 0.26, *K* = 7.71), and Anhui (*Hd* = 0.22, *K* = 6.00) exhibited lower diversity. This reduction compared with all-species diversity reflects the exclusion of *P. maculata* (H4) and *P. occulta* (H9) haplotypes from these sites. Neutrality tests for *P. canaliculata* revealed population-specific deviations from neutrality. Fu’s Fs values were significantly positive in Zhejiang (13.61), Shanghai (36.59), and all three Shanghai sub-populations (*P* < 0.02), suggesting an excess of intermediate-frequency alleles consistent with balancing selection or population structure. Tajima’s D was significantly positive in Shanghai overall (*P* < 0.01) and in the Eastern sub-population (*P* < 0.05), indicating non-neutral evolutionary patterns. In contrast, the Anhui population exhibited significantly negative values for Fu’s Fs (*P* < 0.02), Fu and Li’s D and F (*P* < 0.01), reflecting an excess of low-frequency variants indicative of recent population expansion or a founder effect.

Lastly, when looking at the population structure of all *Pomacea* populations, we found that the pairwise F_ST_ estimates revealed substantial variation in genetic differentiation among *Pomacea* populations across East China (Table 3). The overall F_ST_ across all populations was 0.24, indicating moderate genetic differentiation in the region. The highest levels of genetic structure were observed between Jiangsu and the Northern Islands (*F*_ST_ = 0.59) and between Jiangsu and East Shanghai (0.53), indicating limited gene flow among these populations. Similarly, the Northern Islands and East Shanghai populations were moderately to highly differentiated from both Anhui (0.37 and 0.30, respectively) and Zhejiang (0.30 and 0.27, respectively). In contrast, there was minimal genetic differentiation observed between Zhejiang and West Shanghai (0.00), and between Zhejiang and Anhui (0.00), suggesting substantial gene flow or a shared demographic history (Table 3). Among the Shanghai sub-populations, East Shanghai and the Northern Islands populations showed low differentiation (0.12), whereas both were more distinct from West Shanghai (0.26–0.32). This pattern is consistent with the overall sampling, which emphasized high-density and genetically stable populations in Shanghai, where *Pomacea* currently poses the greatest invasion risk. Consequently, the strongest resolution of genetic structure is observed among the Shanghai sub-populations, whereas the comparatively low and uneven sample numbers from Anhui, Jiangsu, and Zhejiang may not fully capture within-province diversity. Instead, these provincial populations primarily provide regional context for evaluating broader spatial differentiation relative to Shanghai.

**Table 3:** Estimation matrix of pairwise population fixation index (F_ST_) comparisons of genetic differentiation among all *Pomacea* populations in East China using the *COI* (623 bp)

	Anhui	Jiangsu	Zhejiang	Shanghai	W. Sh	E. Sh	N. Is
Anhui	–	0.04	0.00	0.14	0.04	0.30	0.37
Jiangsu	0.04	–	0.22	0.38	0.27	0.53	0.59
Zhejiang	0.00	0.22	–	0.08	0.00	0.27	0.30
Shanghai	0.14	0.38	0.08	–	0.09	0.06	0.12
W. Sh	0.04	0.27	−0.00	0.09	–	0.26	0.32
E. Sh	0.30	0.53	0.27	0.06	0.26	–	0.12
N. Is	0.37	0.59	0.30	0.12	0.32	0.12	–
Overall F_ST_ = 0.24, Overall Nm = 0.77							

Shanghai sub-populations are as follows, West Shanghai (W. Sh), East Shanghai, (E. Sh) and Northern Islands (N. Islands)

## Discussion

### Risk of *Angiostrongylus* in East China

Human angiostrongyliasis has emerged as a significant public health concern in China, particularly following major outbreaks across the country, most of which were linked to the consumption of infected *Pomacea* snails sold in local markets [14]. Although regulatory efforts have since improved, recent sporadic cases of human angiostrongyliasis indicate that surveillance remains inadequate, particularly in newly colonized regions where public awareness is low and diagnostic capacity is limited [16, 47, 48]. Our survey revealed variation in *Pomacea* density across sites in Anhui, Jiangsu, Shanghai, and Zhejiang, largely driven by habitat type and local environmental conditions. High-density populations were concentrated in mostly stagnant or slow-flowing habitats such as urban ponds, drainage canals, and wetlands, where macrophyte cover, detritus accumulation, and shallow water provided favorable conditions for feeding and egg-laying. These patterns align with previous studies showing that *Pomacea* abundance increases in sheltered, vegetation-rich habitats [49, 50]. Conversely, low densities or sites without *Pomacea* occurred in lakes, river channels, and parks with frequent human management, where higher flow, greater water depth, or limited vegetation reduce habitat suitability [49]. Moreover, local habitat modification, such as vegetation removal and the use of molluscicides, also contributes to the number of sites with low or absent *Pomacea* populations [51]. These density patterns are epidemiologically important because areas supporting persistent, high-density populations present a higher likelihood of sustaining *A. cantonensis* transmission should the parasite be introduced.

In addition, our survey found that none of the *Pomacea* snails examined were infected with *A. cantonensis*. Although the lung microscopy method found some individuals from the Fengxian, Minhang, and Pudong New Area districts of Shanghai that had abnormal structures in their lung sacs. However, subsequent testing using both high-stringency PCR and LAMP assays confirmed that these samples were not infected, as no *A. cantonensis* DNA was detected in any of the snails. Therefore, these abnormal structures were likely caused by other pathogenic processes, such as microbial infection, or immune encapsulation of foreign material. For example, Guo et al. [52] reported nodule-like structures in the lung sac of *P. canaliculata* caused by other microbial pathogens that closely resembled those induced by *A. cantonensis*, highlighting the risk of misidentifying infection based on morphology alone. Although the exact cause could not be determined, these lesions most likely reflect general host–pathogen interactions or nonspecific stress responses rather than early-stage *A. cantonensis* infection. To further confirm this interpretation, we note that both the *ITS2* PCR and *COI*-based LAMP assays used here are highly sensitive, capable of detecting 100 picograms to 1 femtogram of target DNA, sufficient to detect a single *A. cantonensis* larva [19, 21, 53]. Given this sensitivity, the negative results are most consistent with a true absence of *A. cantonensis* infection in the sampled snails rather than assay failure, underscoring the value of multiple diagnostic methods for minimizing both false negatives and false positives in surveillance for emerging parasitic infections.

Previous parasitological surveys of *A. cantonensis*-infected gastropods have primarily focused on the southern provinces of China, where the parasite is considered highly endemic [16, 53–58]. However, several studies have found *A. cantonensis*-infected gastropods outside of the endemic range of southern China. For example, Lv et al. [9] identified *A. cantonensis*-infected *Pomacea* snails as far north as Taizhou City in the Zhejiang Province. In addition, recent field investigations in Sichuan Province [59] and Anhui Province [18] provide new evidence of northward expansion. In these studies, *P. canaliculata* were found naturally infected for the first time in Zigong (Sichuan) and Ma’anshan (Anhui), confirming the presence of *A. cantonensis* in both regions. Moreover, predictive modelling has shown a substantial expansion of the parasite’s endemic range in mainland China, with over 600,000 km^2^ projected as suitable by the 2020s and more than 1.3 million km^2^ by the 2030s, primarily in the northern and eastern provinces [15]. This projected northward expansion includes currently nonendemic provinces such as Jiangsu and Shanghai, where rising temperatures and the widespread establishment of *Pomacea* snails may create suitable conditions for *A. cantonensis* development and transmission. Although no *A. cantonensis* infections were detected in the present survey, these negative results should not be interpreted as the complete absence of risk. Low-prevalence infections may remain undetected due to spatial and temporal variability, and the widespread presence of highly compatible intermediate hosts such as *P. canaliculata* underscores the need for continued, sensitive molecular surveillance. Moreover, it is important to note that our sampling was conducted during a single summer–autumn period, when *Pomacea* activity and detectability are highest. Seasonal shifts in snail density, and reproduction may influence parasite exposure and detection probability, particularly during cooler months when activity declines [16]. Future multi-season sampling would help capture these temporal dynamics and more accurately link infection risk with *Pomacea* population patterns in Shanghai.

### Genetic diversity of *Pomacea* populations in East China

The genetic diversity of *Pomacea* populations in Shanghai and East China reflects a complex invasion history shaped by multiple introduction events, high genetic diversity, and variable gene flow across the region. Our results show that population structure is influenced by both regional connectivity and localized isolation. Shanghai exhibits the highest diversity, together with strongly positive neutrality test values, indicating the coexistence of multiple introduced lineages and underlying population structure driven by repeated human-mediated dispersal. In contrast, the significantly negative neutrality values observed in Anhui are consistent with recent population expansion following a founder event. The lack of variation in Jiangsu and its strong F_ST_ isolation from other populations likely reflects limited hydrological connectivity and fewer introduction pathways, whereas the low differentiation between Zhejiang, Anhui, and western Shanghai suggests active gene flow facilitated by shared waterways and human transport networks. Together, these patterns demonstrate that both natural landscape features and anthropogenic movement shape the spatial genetic structure of *Pomacea* across East China. This study is the first to analyze multiple *Pomacea* populations within Shanghai and its neighboring provinces, building upon earlier nationwide studies that lacked fine-scale regional resolution [5, 6, 10]. For example, Wei et al. [10] used both mitochondrial (*COI*) and nuclear (ITS1) markers to characterize country-wide population structure in *P. canaliculata*, but noted that the ITS1 marker evolves too slowly to resolve fine-scale differentiation in recently established invasive populations. While our *COI*-based analyzes provide high-resolution insights into local invasion dynamics, *COI* only shows the maternal lineage and cannot reflect biparental processes such as contemporary gene flow, recombination, or hybridization. Although *COI* remains a powerful marker for species identification and haplotype resolution, its inherent limitations underline the need for complementary nuclear data. Future work integrating faster-evolving nuclear markers (e.g., whole-genome sequencing) will enable a more comprehensive understanding of genetic structure, introgression, and invasion history in East China [12].

Our findings align with previous research showing substantial mitochondrial diversity in *Pomacea* populations across East Asia [4, 35–39, 44, 59, 60]. Our results also show that only a single lineage of both *P. maculata* and *P. occulta* appears to be established in East China, suggesting more limited introduction events compared with *P. canaliculata*. Analysis of *COI* haplotype distribution revealed 25% of populations were monomorphic, which is characteristic of newly colonized areas founded by a small number of individuals. Meanwhile, other sites displayed moderate-to-high haplotype diversity and low genetic structure, especially between Anhui, Jiangsu, Zhejiang, and western Shanghai, indicating substantial gene flow and regional connectivity. The most common haplotypes in our study (e.g., H1, H3, and H5) have been repeatedly documented in South and West China [5, 10], likely representing earlier or regionally dominant introduction events. These lineages may have spread inland from southern provinces through both natural dispersal methods (e.g., birds and flooding) or human-mediated pathways (e.g., transport and trade). Conversely, the unique haplotypes only found in Shanghai (H5–H8) provide important insight into the invasion dynamics of *P. canaliculata* in East China. The presence of these unique haplotypes suggests recent introductions or ongoing local diversification. Shanghai’s role as a major commercial trade and transportation hub provides multiple opportunities for inadvertent introduction of *Pomacea* snails or eggs. Additional pathways such as the aquarium trade and the movement of aquatic plants are also known sources of *Pomacea* introductions [61]. Moreover, comparisons with international *COI* datasets show that these haplotypes cluster with sequences from Argentina, Uruguay, Japan, the Philippines, and several regions of China, suggesting a combination of direct introductions from the native South American range and secondary introductions via Southeast Asia through trade-mediated pathways [4, 10, 39, 41, 42]. These patterns reinforce *P. canaliculata* as the dominant invasive species in China and that its current distribution reflects multiple, independent introduction events likely facilitated by international maritime trade. Similarly, the single *P. maculata* haplotype (H4) was only found in Shanghai, clustering with sequences from West China, Malaysia, and Singapore, indicating a more limited but regionally connected introduction history. Likewise, the *P. occulta* haplotype (H9) only found in Anhui matched sequences from southern and eastern China, consistent with its known native distribution in southern China.

The ongoing influx of *Pomacea* into Shanghai from external sources raises concerns about the potential introduction of *A. cantonensis* and other foreign pathogens associated with invasive species [2, 55]. Despite the current absence of *A. cantonensis* in Shanghai and Jiangsu, the detection of *COI* haplotypes shared with endemic populations, coupled with strong gene flow from southern regions, suggest that repeated human-mediated transport could readily introduce the parasite into East China [62, 63]. International trade (e.g., Brazil, Indonesia, Thailand) and domestic trade (e.g., Guangdong, Fujian, Hainan) may facilitate the spread of the parasite. This can occur through the movement of infected rodents, snails, and other hosts. Moreover, given the ecological adaptability of *Pomacea* snails and the broad intermediate host range of *A. cantonensis*, even isolated introductions of infected rodents or gastropods could trigger local transmission. Climate-driven range expansion is also expected to push *A. cantonensis* into more temperate regions such as Shanghai and Jiangsu, with ecological niche models projecting a poleward shift in suitable habitat despite an overall global decline in highly suitable areas [64]. Therefore, in order to mitigate this risk, proactive measures, including strengthened port-of-entry biosecurity and targeted surveillance along major trade and transport corridors, will be critical [65]. Without such interventions, the establishment of *A. cantonensis* in currently nonendemic regions such as Shanghai may become increasingly likely or inevitable. Once introduced, newly endemic areas could pose substantial threats to agriculture and public health, particularly where highly compatible invasive hosts such as *Pomacea* are already widespread.

Lastly, several limitations of this study should be acknowledged. First, sampling was conducted over a single year, which may not fully capture interannual or seasonal variation in *Pomacea* population dynamics or *A. cantonensis* transmission intensity in East China. Second, although multiple sensitive diagnostic approaches were employed, infections occurring at very low prevalence, in highly localized foci, or at early developmental stages may not have been detected. Third, *Pomacea* population genetic analyses relied on mitochondrial *COI* markers, which represent only maternal inheritance and cannot resolve recent gene flow, recombination, or hybridization. Future studies incorporating multi-year and multi-season sampling, coupled with expanded molecular screening and nuclear genomic data, would provide a more comprehensive understanding of invasion dynamics, population connectivity, and transmission risk.

## Conclusions

In this study, no *A. cantonensis-*infected *Pomacea* snails were detected in Shanghai or the surrounding East China sites. However, this finding does not rule out the possibility of undetected, low-prevalence infections in Shanghai and other nonendemic regions of East China. Moreover, the frequent introductions and continued establishment of genetically diverse *Pomacea* populations across the East China region highlight a growing ecological and public health risk. Therefore, continued surveillance, public education, and improved biosecurity protocols are essential. These measures can help prevent the future expansion of *A. cantonensis* into Shanghai and other East China provinces. Importantly, our findings highlight the need to move beyond traditional surveys and incorporate genetic and molecular xenomonitoring approaches to more effectively anticipate and detect emerging zoonotic threats in nonendemic regions.

## Supplementary Information

Supplementary Material 1: Table S1 All *Pomacea* collection sites information; Table S2 Lung dissections and molecular screening results; Table S3 PCR and LAMP primer information; Table S4 Haplotype composition for each site; Table S5 NCBI *COI* reference sequences; Figure S1 Laboratory procedures; Figure S2 Specificity of the Anc-*ITS2* PCR assay; Figure S3 PCR and LAMP assay results; and Figure S4 Anc-*COI* LAMP primer binding sites.

## Data Availability

The datasets supporting the findings of this article are included within the paper. The COI sequences of the genotyped *Pomacea* snails in this study are available at GenBank accession numbers PV793382–PV793390.
