# Supplementary material for: Population genetic diversity of invasive Pomacea snails and surveillance of Angiostrongylus cantonensis in Shanghai, East China
Source: Parasit Vectors. 2026 Mar 9;19:162. doi: 10.1186/s13071-025-07224-w (PMC13085369; doi:10.1186/s13071-025-07224-w)
Supplement: Supplementary file 1 — Supplementary Material 1: Table S1 All Pomacea collection sites information; Table S2 Lung dissections and molecular screening results; Table S3 PCR and LAMP primer information; Table S4 Haplotype composition for each site; Table S5 NCBI COI reference sequences; Figure S1 Laboratory procedures; Figure S2 Specificity of the Anc-ITS2 PCR assay; Figure S3 PCR and LAMP assay results; and Figure S4 Anc-COI LAMP primer binding sites. [file 13071_2025_7224_MOESM1_ESM.docx]

**Table S1.** Summary of *Pomacea* snail collection sites in East China

| Anhui | | | | | | | |
| --- | --- | --- | --- | --- | --- | --- | --- |
|  | Date | Area | Site | *Pomacea* | Coordinates | Density | Code |
| **A1** | **16/11/2024** | **Hefei** | **Luogang Park** | **Present** | **31.7843, 117.3057** | **High** | **HAN** |
| A2 | 16/11/2024 | Hefei | Motan Wetland | Present | 31.7768, 117.3809 | Low | - |
| A3 | 17/11/2024 | Chaohu | Zhegao Wetland | Present | 31.6246, 117.7828 | Low | - |
| A4 | 17/11/2024 | Hefei | Chaohu Hubin Wetland | Absent | 31.7182, 117.3724 | Zero | - |
|  | | | | | | | |
| Jiangsu | | | | | | | |
|  | Date | Area | Site | *Pomacea* | Coordinates | Density | Code |
| J1 | 01/08/2024 | Suzhou | Wusong Jiang | Present | 31.2873, 120.9070 | Low | - |
| J2 | 01/07/2024 | Suzhou | Taihu Wetland | Present | 31.3227, 120.3570 | Low | - |
| J3 | 01/07/2024 | Wuxi | Lihu Wetland | Present | 31.5182, 120.2471 | Low | - |
| J4 | 01/08/2024 | Wuxi | Lihu Wetland | Present | 31.5113, 120.2465 | Low | - |
| J5 | 01/07/2024 | Wuxi | Jinchengwan Park | Present | 31.5204, 120.2864 | Low | - |
| J6 | 20/04/2024 | Zhangjiagang | Jiyang Wetland | Absent | 31.8442, 120.5231 | Zero | - |
| **J7** | **16/07/2024** | **Zhangjiagang** | **Xinsigang River** | **Present** | **31.8676, 120.5013** | **High** | **ZJG** |
| J8 | 01/08/2024 | Nanjing | Jiangjunshan Area | Present | 31.9314,118.7464 | Low | - |
| J9 | 22/02/2025 | Shuangshan Island | Shuangshan Island | Absent | [31.9864, 120.4084](https://www.bing.com/maps?q=Shuangshan+Island+Jiangsu+map&FORM=HDRSC6&cp=31.985711~120.408804&lvl=16.0) | Zero | - |
| J10 | 01/08/2024 | Nanjing | Qilin Park | Present | 32.0176, 118.8913 | Low | - |
| J11 | 15/03/2025 | TaiZhou | Dongcheng River | Absent | 32.4914, 119.9275 | Zero | - |
| J12 | 01/07/2024 | Huai'an | Hongzehu Wetland | Present | 33.2486, 118.3287 | Low | - |
|  | | | | | | | |
| Shanghai | | | | | | | |
|  | Date | Area | Site | *Pomacea* | Coordinates | Density | Code |
| S1 | 01/11/2024 | Fengxian | Zhelin Town | Present | 30.8387, 121.4675 | Low | - |
| S2 | 01/11/2024 | Fengxian | Xingtuan | Present | 30.8445, 121.3912 | Low | - |
| **S3** | **25/09/2024** | **Pudong** | **Huijiao Village** | **Present** | **30.8809, 121.8560** | **High** | **PD2** |
| **S4** | **01/11/2024** | **Fengxian** | **Jinhai** | **Present** | **30.8870, 121.5017** | **High** | **FX51** |
| S5 | 01/11/2024 | Fengxian | Xinfeng | Present | 30.9503, 121.6460 | Low | - |
| **S6** | **01/11/2024** | **Fengxian** | **Xinye Village** | **Present** | **30.9604, 121.3723** | **High** | **FX43** |
| **S7** | **01/10/2024** | **Pudong** | **Chezhan Village** | **Present** | **30.9653, 121.7475** | **High** | **PD5** |
| S8 | 01/11/2024 | Fengxian | Zhanglang | Present | 30.9682, 121.5406 | Low | - |
| **S9** | **01/11/2024** | **Fengxian** | **Meiyuan Village** | **Present** | **30.9885, 121.5984** | **High** | **FX12** |
| S10 | 01/11/2024 | Qingpu | Qiansheng Village | Present | 31.0254, 120.9807 | Low | - |
| **S11** | **01/11/2024** | **Qingpu** | **Liansheng Community** | **Present** | **31.0402, 120.9213** | **High** | **QI5** |
| **S12** | **01/10/2024** | **Pudong** | **Sizao River** | **Present** | **31.0746, 121.6655** | **High** | **PD9** |
| S13 | 01/11/2024 | Qingpu | Wangxian Village | Present | 31.1271, 120.9057 | Low | - |
| S14 | 01/10/2024 | Pudong | Chuanzhou | Present | 31.1416, 121.6153 | Low | - |
| **S15** | **02/09/2024** | **Minhang District** | **Meihang Park** | **Present** | **31.1450, 121.3688** | **High** | **MNP** |
| S16 | 01/10/2024 | Pudong | Changjiegang | Present | 31.1625, 121.7098 | Low | - |
| S17 | 28/05/2024 | Pudong | Expo Culture Park | Present | 31.181, 121.471 | Low | - |
| **S18** | **01/11/2024** | **Qingpu** | **Songshan Village** | **Present** | **31.1955, 121.2425** | **High** | **QI4** |
| S19 | 21/10/2024 | Xuhui | Xujiahui Park | Absent | 31.1990, 121.4381 | Zero | - |
| **S20** | **01/11/2024** | **Qingpu** | **Hui Long Village** | **Present** | **31.2115, 121.1709** | **High** | **QI3** |
| S21 | 12/09/2024 | Pudong | Century Park | Absent | 31.2179, 121.5492 | Zero | - |
| S22 | 11/05/2024 | Putuo | Suzhou Creek | Absent | 31.221, 121.394 | Zero | - |
| S23 | 11/05/2024 | Putuo | Changfeng Park | Absent | 31.2233, 121.3985 | Zero | - |
| S24 | 14/08/2024 | Huangpu | Guangchang Park | Absent | 31.2270, 121.4681 | Zero | - |
| S25 | 04/08/2024 | Putuo | Mengqing Garden | Present | 31.2526, 121.4356 | Low | - |
| **S26** | **08/04/2024** | **Putuo** | **Zhenru Park** | **Present** | **31.254, 121.395** | **High** | **ZHN** |
| **S27** | **04/09/2024** | **Yangpu** | **Yangpu Park** | **Present** | **31.2812, 121.5365** | **High** | **YPP** |
| **S28** | **20/05/2024** | **Yangpu** | **Sipping Park** | **Present** | **31.2874, 121.5061** | **High** | **SPP** |
| S29 | 04/09/2024 | Yangpu | Huangxing Park | Absent | 31.2929, 121.5293 | Zero | - |
| S30 | 01/10/2024 | Pudong | Gaodong Park | Present | 31.3077, 121.6431 | Low | - |
| **S31** | **07/09/2024** | **Baoshan District** | **Gucun Park** | **Present** | **31.3408, 121.3738** | **High** | **GCP** |
| **S32** | **01/10/2024** | **Chongming** | **Hengsha Island** | **Present** | **31.3522, 121.8551** | **High** | **CM2** |
| S33 | 01/11/2024 | Chongming | Qianxin | Present | 31.3851, 121.7201 | Low | - |
| S34 | 21/05/2024 | Chongming | Shangshi Farm | Present | 31.5144, 121.9036 | Low | - |
| S35 | 01/11/2024 | Chongming | Zhongxing Town | Present | 31.5218, 121.7620 | Low | - |
| **S36** | **01/10/2024** | **Chongming** | **Panlong** | **Present** | **31.5928, 121.4659** | **High** | **CM3** |
| **S37** | **01/10/2024** | **Chongming** | **Gangdong** | **Present** | **31.6957, 121.4348** | **High** | **CM5** |
|  | | | | | | | |
| Zhejiang | | | | | | | |
|  | Date | Area | Site | *Pomacea* | Coordinates | Density | Code |
| **Z1** | **09/06/2024** | **Hangzhou** | **Xixi Wetland** | **Present** | **30.2704, 120.0730** | **High** | **HZU** |
| Z2 | 10/06/2024 | Hangzhou | East Lake | Present | 30.2339, 120.1295 | Low | - |

Note: *Pomacea* density was categorized as: Zero (no *Pomacea* snails or egg clutches present), Low (presence of egg clutches and/or juvenile snails only), and High (more than 10 adult *Pomacea* snails present).

**Table S2.** Summary of *Pomacea* snail dissection results and molecular screening for *Angiostrongylus cantonensis* infection.

|  | | **Lung dissections** | | **DNA extracts** | |
| --- | --- | --- | --- | --- | --- |
|  | Site | Nodules present? | Abnormal samples | PCR Positive  (Anc-*ITS2*) | LAMP Positive  (Anc-*COI*) |
| **Anhui** | HAN | 0 /20 | 0 /20 | 0 /20 | - |
| **Jiangsu** | ZJG | 0 /22 | 0 /22 | 0 /20 | - |
| **Shanghai** | CM2 | 0 /38 | 0 /38 | 0 /20 | - |
|  | CM3 | 0 /60 | 0 /60 | 0 /20 | - |
|  | CM5 | 0 /60 | 0 /60 | 0 /20 | - |
|  | FX12 | 0 /40 | 1 /40 | 0 /12 | - |
|  | FX43 | 0 /22 | 0 /22 | 0 /12 | - |
|  | FX51 | 0 /41 | 0 /41 | 0 /12 | - |
|  | GCP | 0 /18 | 0 /18 | 2 /18 * | 0 /18 |
|  | MNP | 0 /20 | 1 /20 | 3 /20 * | 0 /20 |
|  | PD2 | 0 /100 | 2 /100 | 0 /20 | - |
|  | PD5 | 0 /49 | 3 /49 | 0 /20 | - |
|  | PD9 | 0 /96 | 3 /96 | 0 /20 | - |
|  | QI3 | 0 /12 | 0 /12 | 0 /12 | - |
|  | QI4 | 0 /12 | 0 /12 | 0 /12 | - |
|  | QI5 | 0 /12 | 0 /12 | 0 /12 | - |
|  | SPP | 0 /20 | 0 /20 | 0 /20 | - |
|  | YPP | 0 /20 | 0 /20 | 0 /20 | - |
|  | ZHN | 0 /20 | 0 /20 | 0 /20 | - |
| **Zhejiang** | HZU | 0 /18 | 0 /18 | 0 /18 | - |
|  | | | | | |
| **Total** | | 0 /700 | 10 /700 | 5 /348 * | 0 /38 |

Note: *All Anc-*ITS2* PCR–positive samples were shown to be non-specific amplifications, which disappeared when the annealing temperature was increased from 50°C to 55°C.

**Table S3.** PCR and LAMP primer information

| Assay | Primer | Primer Sequence (5' - 3') | Length | Annealing | Reference |
| --- | --- | --- | --- | --- | --- |
| Pom-*COI*  PCR | LCO1490 | GGT CAA CAA ATC ATA AAG ATA TTG G | 25 | 50°C | Folmer et al. [15] |
|  | HCO2198 | TAA ACT TCA GGG TGA CCA AAA AAT CA | 26 |  |  |
|  | | | | |  |
| Anc-*ITS2*  PCR | Anc-*ITS2*-F | ACG TCT GGT TCA GGG TTG TT | 20 | 50°C (Low) & 55°C (High) | Jiang et al. [16] |
|  | Anc-*ITS2*-R | TTA GTT TCT TTT CCT CCG CT | 20 |  |  |
|  | | | | |  |
| Anc-*COI*   Lamp | *COI* -F3 | AGA CAG TCT ACT TTA TAT TTA ACG G | 25 | 65°C | **This Study** |
|  | *COI* -B3 | TAA AGT TGC CAA CCA CCT | 18 |  |  |
|  | *COI* -FIP | GCC CAA ACC ACA CAA CCA ATC GGT TAT TTG GGT ATG GTT TAT GC | 44 |  |  |
|  | *COI* -BIP | ACG GTT GGT ATG GAT TTA GAT TCT CAA AAA CCT TCA CTC CCG TA | 44 |  |  |
|  | *COI* -LB | TAC TGC GGC TAC TAT AGT TAT TGC G | 25 |  |  |

**Table S4.** Haplotype composition and diversity indices of East China sampling sites.

|  | Hd | π | *Pomacea canaliculata* | | | | | | | *P. maculata* | *P. occulta* |
| --- | --- | --- | --- | --- | --- | --- | --- | --- | --- | --- | --- |
|  |  |  | H1 | H2 | H3 | H5 | H6 | H7 | H8 | H4 | H9 |
| CM2 | 0.467 | 0.02 | 3 | 0 | 7 | 0 | 0 | 0 | 0 | 0 | 0 |
| CM3 | 0.555 | 0.05 | 0 | 0 | 5 | 0 | 0 | 0 | 0 | 5 | 0 |
| CM5 | 0.533 | 0.05 | 0 | 0 | 6 | 0 | 0 | 0 | 0 | 4 | 0 |
| FX12 | 0.533 | 0.05 | 0 | 0 | 0 | 6 | 0 | 0 | 0 | 4 | 0 |
| FX43 | 0.533 | 0.05 | 0 | 0 | 0 | 4 | 0 | 0 | 0 | 6 | 0 |
| FX51 | - | - | 0 | 0 | 0 | 10 | 0 | 0 | 0 | 0 | 0 |
| GCP | - | - | 0 | 0 | 0 | 0 | 0 | 10 | 0 | 0 | 0 |
| HAN | 0.378 | 0.03 | 8 | 1 | 0 | 0 | 0 | 0 | 0 | 0 | 1 |
| HZU | 0.467 | 0.02 | 7 | 0 | 3 | 0 | 0 | 0 | 0 | 0 | 0 |
| MNP | 0.600 | 0.03 | 6 | 1 | 0 | 0 | 0 | 0 | 3 | 0 | 0 |
| PD2 | 0.467 | 0.02 | 7 | 0 | 0 | 3 | 0 | 0 | 0 | 0 | 0 |
| PD5 | 0.556 | 0.03 | 5 | 0 | 0 | 5 | 0 | 0 | 0 | 0 | 0 |
| PD9 | 0.467 | 0.05 | 0 | 0 | 0 | 7 | 0 | 0 | 0 | 3 | 0 |
| QI3 | - | - | 10 | 0 | 0 | 0 | 0 | 0 | 0 | 0 | 0 |
| QI4 | 0.644 | 0.03 | 5 | 1 | 4 | 0 | 0 | 0 | 0 | 0 | 0 |
| QI5 | 0.200 | 0.01 | 9 | 0 | 0 | 0 | 1 | 0 | 0 | 0 | 0 |
| SPP | 0.689 | 0.03 | 5 | 0 | 2 | 0 | 0 | 3 | 0 | 0 | 0 |
| YYP | 0.533 | 0.03 | 4 | 0 | 6 | 0 | 0 | 0 | 0 | 0 | 0 |
| ZHN | - | - | 10 | 0 | 0 | 0 | 0 | 0 | 0 | 0 | 0 |
| ZJG | - | - | 10 | 0 | 0 | 0 | 0 | 0 | 0 | 0 | 0 |

**Table S5.** NCBI reference information for *COI* sequences used for the phylogenetic and haplotype network analysis.

| *P. canaliculata* | | |
| --- | --- | --- |
| Accession no. | Location | Reference |
| AB433759 | Japan | [1] |
| AB433762 | Japan |  |
| AB728574 | Argentina | [2] |
| AB728575 | Argentina |  |
| AB728576 | Argentina |  |
| AB728577 | Argentina |  |
| EU528483 | Philippines | [3] |
| EU528509 | Argentina |  |
| EU528520 | Argentina |  |
| EU528586 | Taiwan, China |  |
| EU528587 | Taiwan, China |  |
| FJ710313 | Uruguay | [4] |
| FJ946824 | Guangdong, China | - |
| MF170059 | Yunnan, China | [5] |
| MG230781 | Malaysia | [6] |
| MH602333 | Jiangxi, China | - |
| MK992488 | Uruguay | [7] |
| MN623431 | Malaysia | [8] |
| MT246816 | China | - |
| MT806215 | Hong Kong, China | [9] |
| MZ381599 | Guangxi Zhuang, China | - |
| MZ396719 | Shanghai, China |  |
| MZ396735 | Jiangsu, China |  |
| ON054159 | Jiangsu, China | - |
| **PV793382 (H1)** | **Shanghai, China** | **This Study** |
| **PV793383 (H2)** | **Shanghai, China** |  |
| **PV793384 (H3)** | **Shanghai, China** |  |
| **PV793386 (H5)** | **Shanghai, China** |  |
| **PV793382 (H6)** | **Shanghai, China** |  |
| **PV793382 (H7)** | **Shanghai, China** |  |
|  |  |  |
|  |  |  |
| *P. maculata* | | |
| Accession no. | Location | Reference |
| FJ946819 | Guangdong, China | - |
| KY081759 | Singapore | [10] |
| MF401379 | Sichuan, China | [11] |
| MG230771 | Malaysia | [12] |
| MH602335 | Jiangxi, China | - |
| MH602335 | Jiangxi, China |  |
| MH602337 | Jiangxi, China |  |
| MT246783 | China | - |
| MT246784 | China |  |
| **PV793385 (H4)** | **Shanghai, China** | **This Study** |
|  |  |  |
| *P. occulta* | | |
| Accession no. | Location | Reference |
| KP310474 | Fujian, China | [13] |
| MK756260 | China | [14] |
| MK756272 | Zhejiang, China |  |
| MT806275 | Hong Kong, China | [9] |
| MT806312 | Hong Kong, China |  |
| MT806335 | Hong Kong, China |  |
| OR477332 | Hainan, China | - |
| PV123600 | China | - |
| PV123610 | China |  |
| PV123620 | China |  |
| **PV793390 (H9)** | **Anhui, Hefei, China** | **This Study** |

**References:**

1. Matsukura K, Okuda M, Kubota K, Wada T. Genetic divergence of the genus *Pomacea* (Gastropoda: Ampullariidae) distributed in Japan, and a simple molecular method to distinguish *P. canaliculata* and *P. insularum*. Appl Entomol Zool. 2008;43(4):535–40.
2. Yoshida K, Matsukura K, Cazzaniga NJ, Wada T. Tolerance to low temperature and desiccation in two invasive apple snails, *Pomacea canaliculata* and *P. maculata*, collected in their original distribution area (northern and central Argentina). J Molluscan Stud. 2014;80(1):62–6.
3. Hayes KA, Joshi RC, Thiengo SC, Cowie RH. Out of South America: multiple origins of non-native apple snails in Asia. Divers Distrib. 2008;14(4):701–12.
4. Hayes KA, Cowie RH, Thiengo SC. A global phylogeny of apple snails: Gondwanan origin, generic relationships, and the influence of outgroup choice (Caenogastropoda: Ampullariidae). Biol J Linn Soc. 2009;98(1):61–76.
5. Cang-Lin ZH, Jia PE, Zhen RA, Jin-Rong ZI, Ya-Ming YA. Genotyping and polymorphism analysis of cytochrome c oxidase subunit I gene of *Pomacea canaliculata* from Lincang City in Yunnan Province. Chin J Schisto Control. 2018;30(2):179. Chinese.
6. Rama Rao S, Liew TS, Yow YY, Ratnayeke S. Cryptic diversity: Two morphologically similar species of invasive apple snail in Peninsular Malaysia. PLoS One. 2018;13(5):e0196582.
7. Glasheen PM, Burks RL, Campos SR, Hayes KA. First evidence of introgressive hybridization of apple snails (*Pomacea* spp.) in their native range. J Molluscan Stud. 2020;86(2):96–103.
8. Kannan A, Rao SR, Ratnayeke S, Yow YY. Efficiency of universal mitochondrial DNA barcodes for species discrimination of *Pomacea canaliculata* and *Pomacea maculata*. PeerJ. 2020;8:e8755.
9. Yang QQ, Ip JC, Zhao XX, Li JN, Jin YJ, Yu XP, et al. Molecular analyses reveal three morphologically similar species of non-native apple snails and their patterns of distribution in freshwater wetlands of Hong Kong. Divers Distrib. 2022;28(1):97–111.
10. Ng TH, Tan SK, Ahmad A, Van Tu D, Joshi RC, Wang WY, et al. Not in the Least Concern: anthropogenic influences on a Southeast Asian apple snail *Pila scutata* (Ampullariidae). Oryx. 2019;53(2):230–8.
11. Yang QQ, Liu SW, Song F, Liu GF, Yu XP. Comparative mitogenome analysis of four apple snail species (Ampullariidae: *Pomacea*). Int J Biol Macromol. 2018;118:525–33.
12. Phoong MJ, Hah HE, Rao SR, Yow YY, Ratnayeke S. Invasive apple snails in wetlands of Selangor, Malaysia: species, distribution, and ecological associations. J Trop Biol Conserv. 2018;15:43–60.
13. Yang Q, Liu S, He C, Cowie RH, Yu X, Hayes KA. Invisible apple snail invasions: importance of continued vigilance and rigorous taxonomic assessments. Pest Manag Sci. 2019;75(5):1277–86.
14. Yang QQ, Yu XP. A new species of apple snail in the genus *Pomacea* (Gastropoda: Caenogastropoda: Ampullariidae). Zool Stud. 2019;58:e13.
15. Folmer O, Hoeh WR, Black MB, Vrijenhoek RC. Conserved primers for PCR amplification of mitochondrial DNA from invertebrates. Mol Mar Biol Biotechnol. 1994;3(5):294–9.
16. Jiang L, Li T, Jiang Y, Liu Y, Chen S, Liu H, et al. A cost-effective strategy for identifying *Angiostrongylus* spp. larvae in *Achatina fulica*: combined morphological and molecular biology. Parasites Vectors. 2025;18:47.


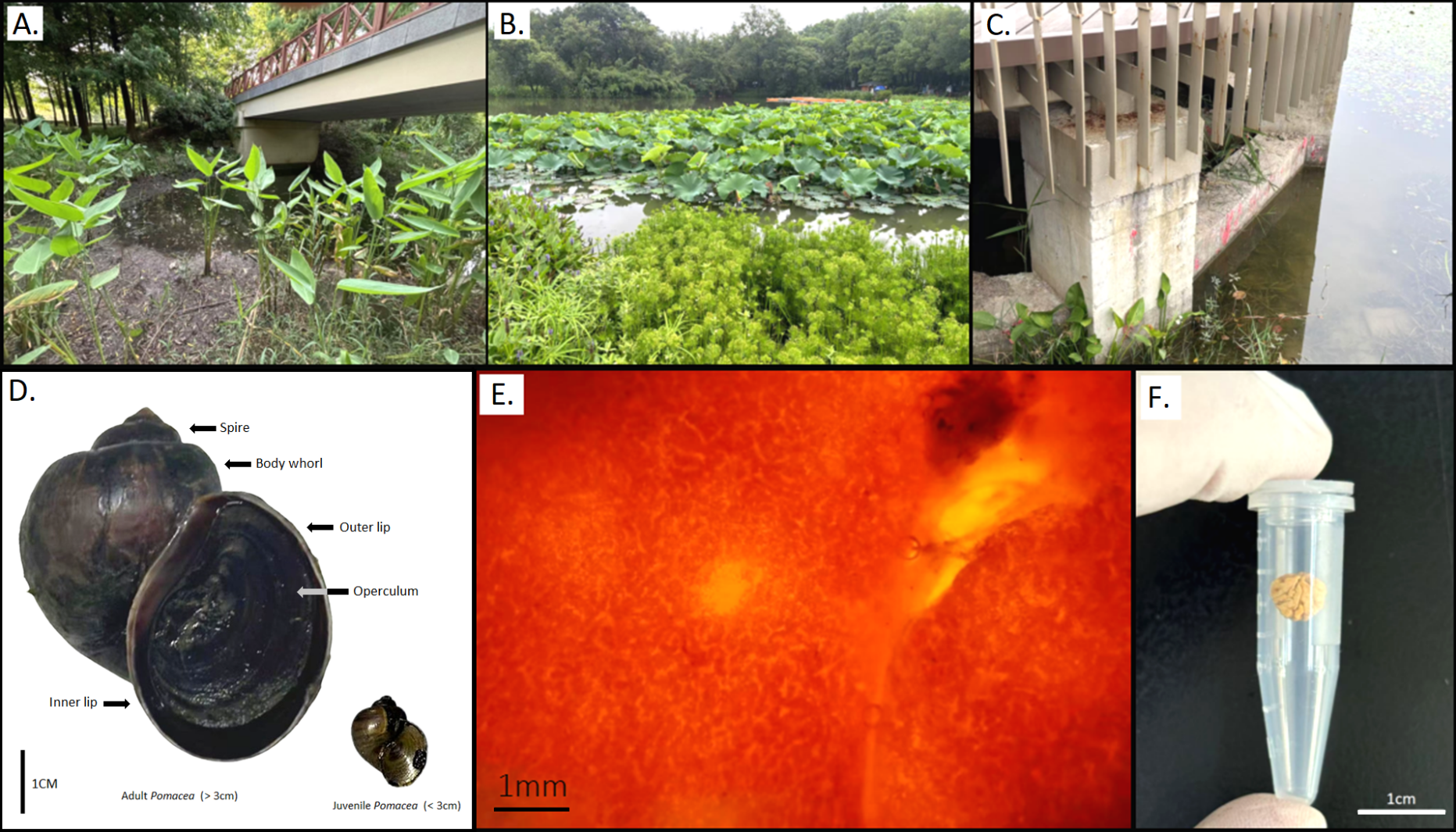


**Figure S1.** Examples of high-density *Pomacea* collection sites, such as Gucun park, Shanghai (A); Xixi wetlands, Zhejiang (B); and Luogang park, Anhui (C). Laboratory procedures showing adult and juvenile *Pomacea* specimens collected from the field (scale bar: 1cm; D), examination of the *Pomacea* lung sac under a light microscope (scale bar: 1mm; E), and *Pomacea* lung tissue collection for DNA extraction (scale bar: 1cm; F).


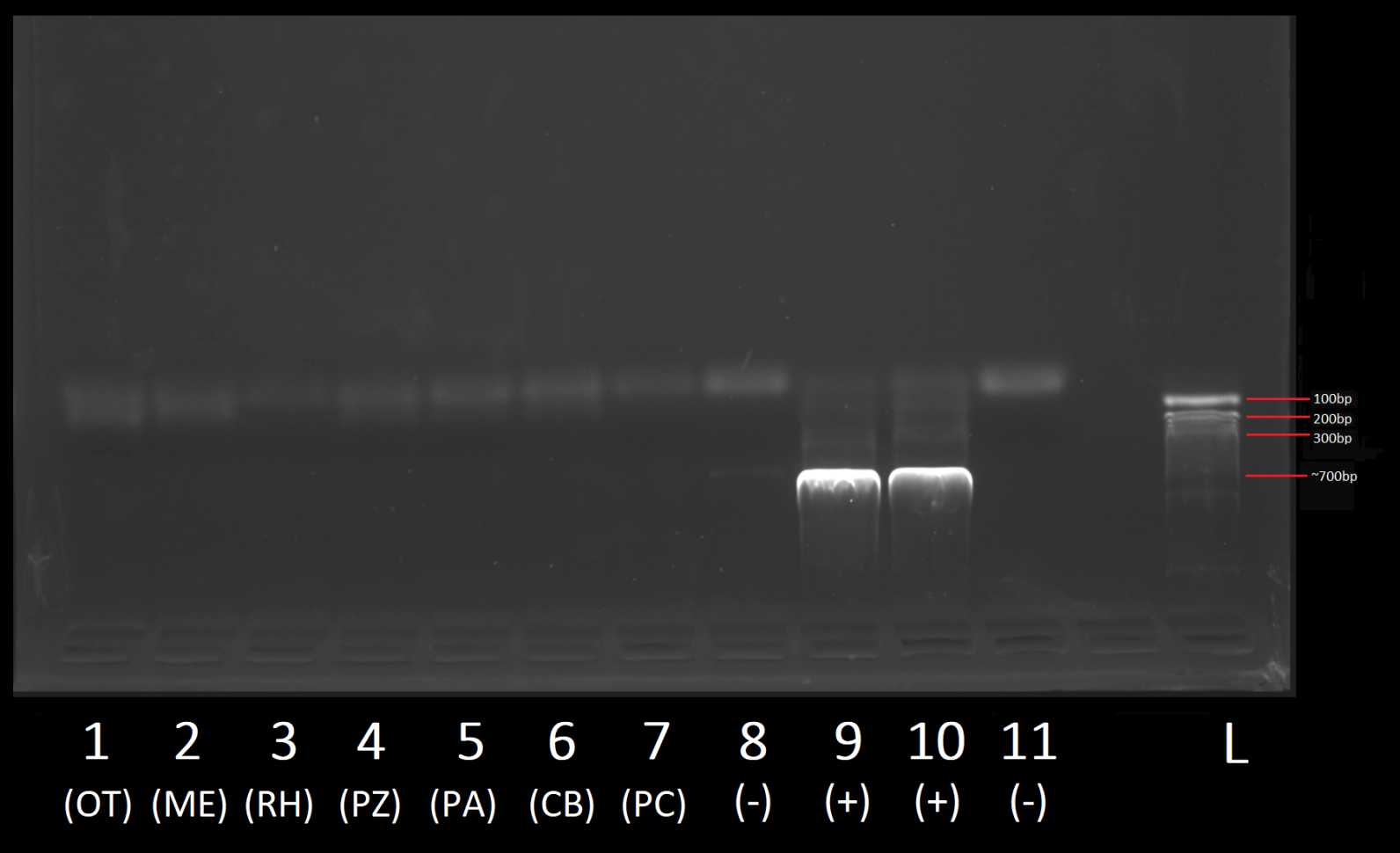


**Figure S2**. Specificity testing of the Anc-*ITS2* PCR (50°C) assay for *Angiostrongylus cantonensis* infection and other gastropod-associated nematodes.
Note: Lane 1 = *Oscheius tipulae* DNA (OT); Lane 2 = Mermithidae sp. DNA (ME); Lane 3 = *Rhabdias* sp. DNA (RH); Lane 4 = *Phasmarhabditis zhejiangensis* DNA (PZ); Lane 5 = Panagrolaimidae sp. DNA (PA); Lane 6 = *Caenorhabditis briggsae* DNA (CB); Lane 7 = uninfected *Pomacea* DNA (PC); Lane 8 = Negative control (Water); Lane 9 = *Angiostrongylus cantonensis*-infected *Pomacea* DNA (+); Lane 10 = *A. cantonensis* DNA (+); Lane 11 = Negative control (Water); Lane L = 100bp Ladder.


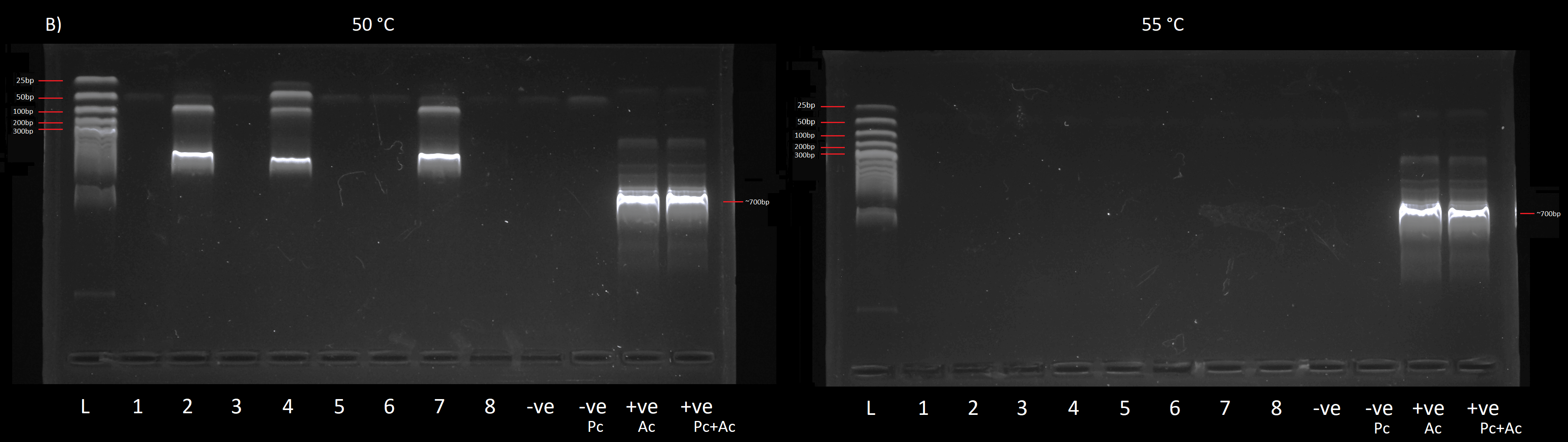

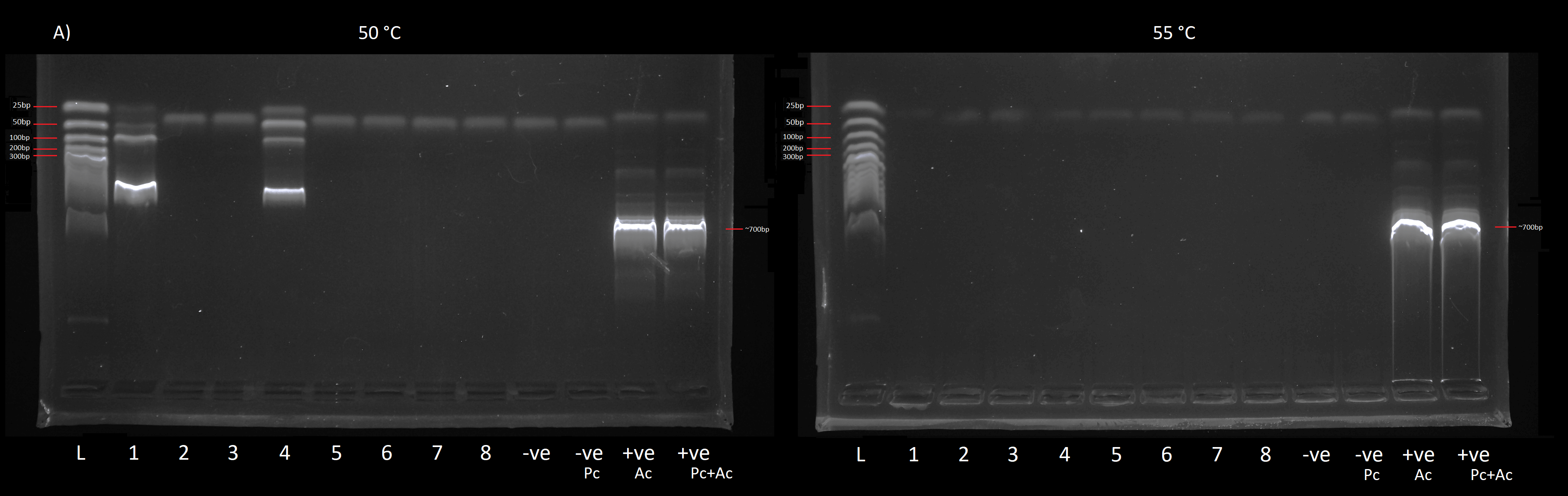


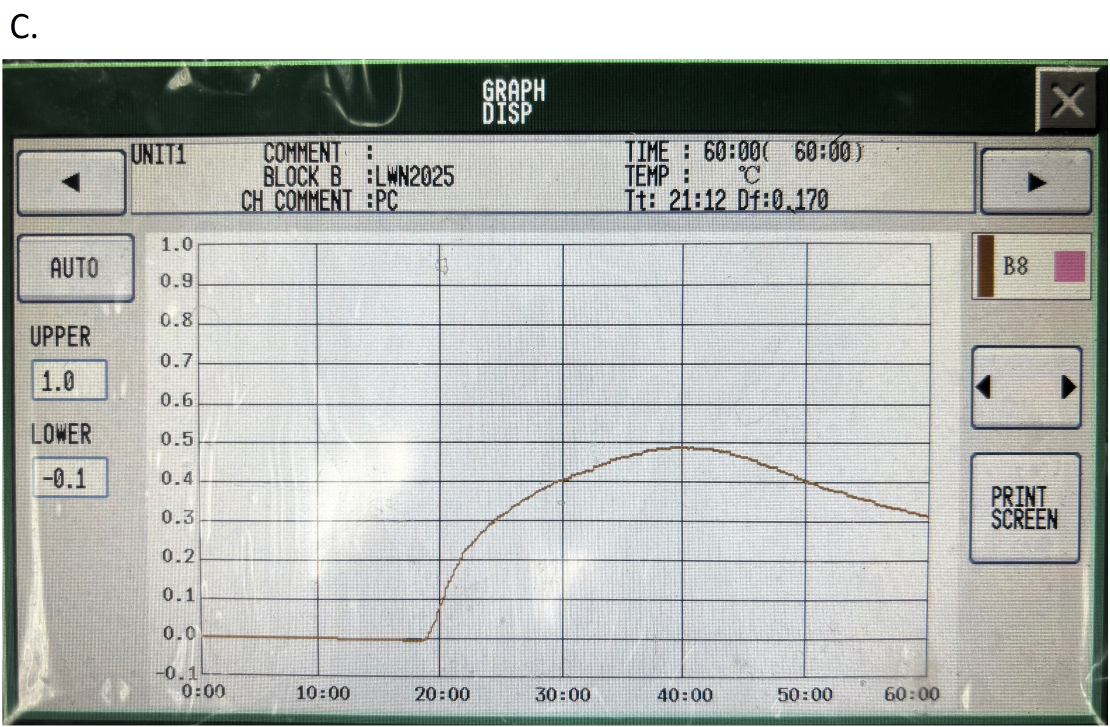


**Figure S3. Detection of *Angiostrongylus cantonensis* DNA in *Pomacea* samples using PCR and LAMP assays.** Agarose gel electrophoresis of PCR products amplified with Anc-*ITS2* primers from snails collected at Gucun Park (A) and Minhang Park (B) at annealing temperatures of 50°C and 55°C. (C) LAMP assay targeting the *A. cantonensis COI* gene (Anc-*COI*) for both GCP and MHP samples. Only the positive control (*A. cantonensis* DNA) produced a detectable turbidity signal (0.49).

Note: For Gel A: Lane L = 100bp DNA ladder; Lanes 1-8 = GCP DNA samples 1-8; Lane −VE = no-template control (water); Lane −VE Pc = negative control (uninfected *Pomacea* DNA); Lane +VE Ac = positive control (only *A. cantonensis* DNA); Lane +VE Pc/Ac = positive control (*A. cantonensis*-infected *Pomacea* DNA). For Gel B: Lane L = 100 bp DNA ladder; Lanes 1–8 = MHP DNA samples 1–8; Lane −VE = no-template control (water); Lane −VE Pc = negative control (uninfected *Pomacea* DNA); Lane +VE Ac = positive control (only *A. cantonensis* DNA); Lane +VE Pc/Ac = positive control (*A. cantonensis*-infected *Pomacea* DNA).


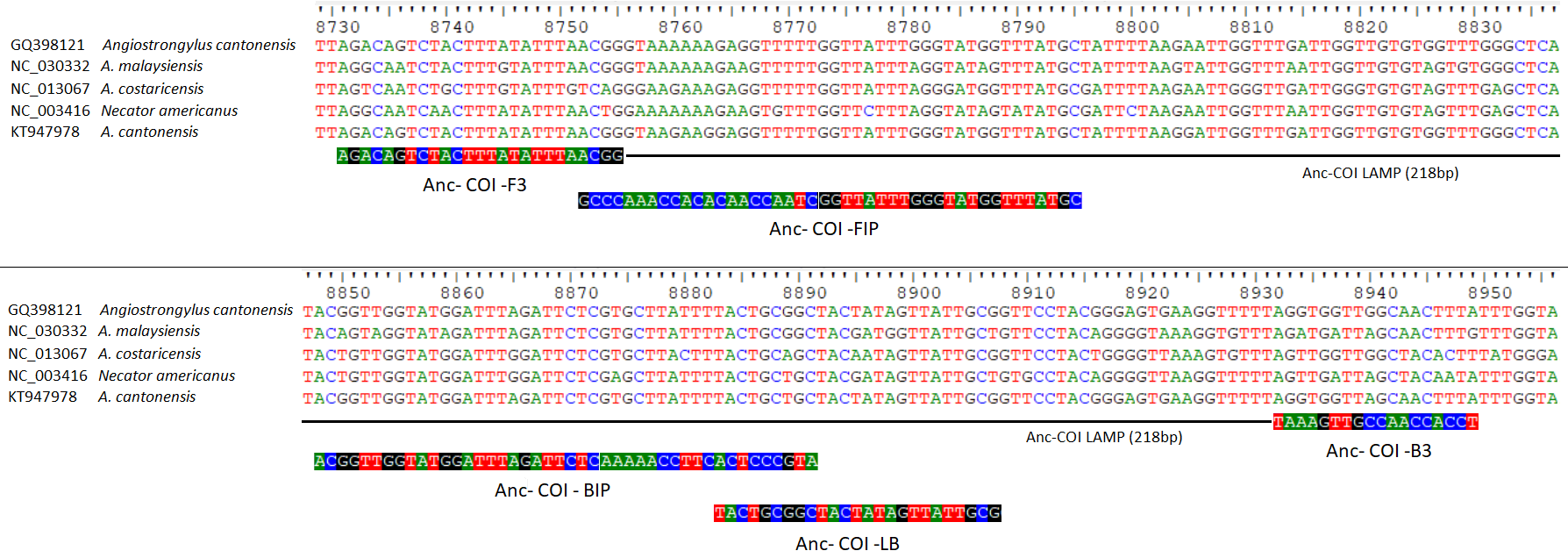


**Figure S4.** Anc-*COI* LAMP primer binding sites and amplified region displayed in BioEdit v5.0.9. LAMP primers included outer primers (F3, B3), inner primers (FIP, BIP), and one loop primer (LB). The assay targets a 218 bp region of the *A. cantonensis COI* gene. Primer specificity was confirmed using complete mitochondrial genomes of *A. cantonensis* and related Metastrongyloidea species, including *A. malaysiensis*, *A. costaricensis*, and *Necator americanus*.
